# Structure and function of therapeutic antibodies approved by the US FDA in 2025

**DOI:** 10.1093/abt/tbag027

**Published:** 2026-05-28

**Authors:** William R Strohl

**Affiliations:** Scientific Advisor Department, BiStro Biotechnology Consulting, 1086 Tullo Farm Rd., 08807 Bridgewater, NJ, United States

**Keywords:** therapeutic antibodies, antibody–drug conjugates (ADCs), bispecific antibodies, new molecular entities, half-life extended antibodies, coagulation pathway, lectin complement pathway, FDA

## Abstract

In 2025, the 50th anniversary of the invention of hybridoma technology, the United States Food and Drug Administration (US FDA) approved a total of 44 new molecular entities (NMEs), of which 10 were therapeutic antibody-based molecules. Additionally, the US FDA approved 31 small molecules (of which one was a peptide), and 3 new nonantibody therapeutic proteins, but no new chimeric antigen receptor (CAR)-T-cell therapeutics. Of the new antibody-based biologics, two were antibody–drug conjugates, one was a T-cell engager–bispecific antibody, two were Fc-modified, half-life extended antibodies, and two targeted the complex coagulation pathway. The eighth novel anti-PD-1 antibody was also approved, along with the newest entry of biologics targeting the neonatal Fc receptor (FcRn). Additionally, two new entries in which hyaluronidase was coformulated with an antibody to provide rapid subcutaneous dosing were FDA-approved. Finally, the first antibody-like scaffold-fusion protein was approved that will compete in an antibody-rich environment.

## Teaser

**Statement of Significance** Ten novel therapeutic antibodies were approved by the US FDA in 2025.

## Introduction–antibodies approved by US FDA in 2025

This year, 2025, marks the 50th anniversary of the first paper, written by Georges Köhler and César Milstein, describing the methodology for generating a monoclonal antibody (mAb) by using their newly created hybridoma technology [1]. Their seminal work, for which they received a Nobel Prize in 1984 [2], essentially marks the beginning of modern-day antibody technologies, which are now so significant in modern medicine. As of 1 April 2026, 169 mAbs and antibody-based biologics have been approved by the United States Food and Drug Administration (FDA) for treating or preventing diseases ranging from Type I diabetes to rheumatoid arthritis to lung cancer [3]. These drugs, valued at over $320 billion (USD) in annual sales in today’s biotechnology market [4], have saved countless lives, improved the quality of life for millions, and have truly revolutionized modern healthcare. All of this essentially founded on the truly ground-breaking work of Köhler and Milstein reported in 1975 [1].

**Figure 1:**
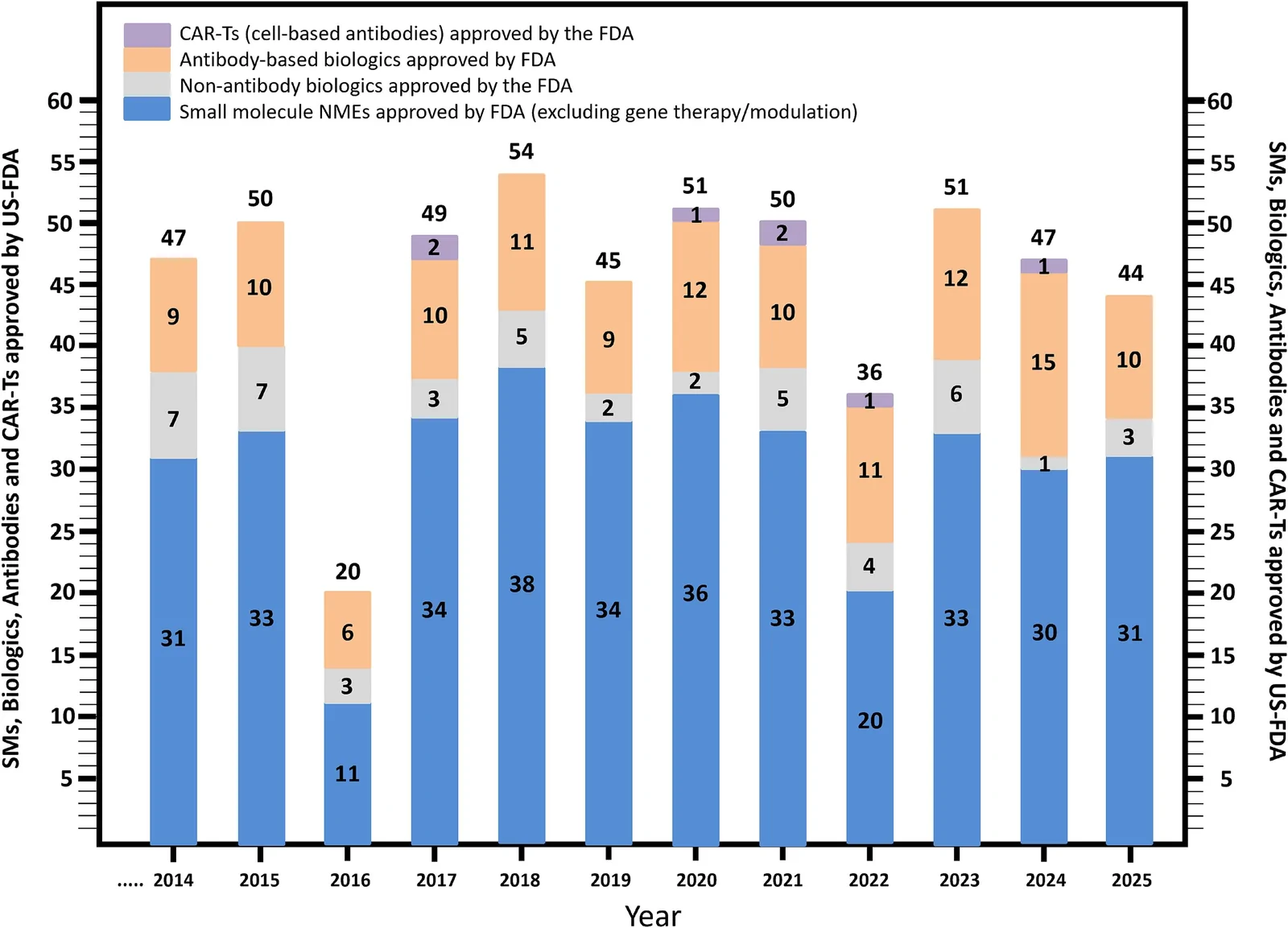
An overview of the FDA approvals for the past 12 years. Number of small molecules, antibody-based biologics, nonantibody recombinant biologics (both Center for Drug Evaluation and Research [CDER] and Center for Biologics Evaluation and Research [CBER]), and chimeric antigen receptor-T cells (CAR-Ts) approved by the US FDA on an annual basis from 2014 to 2025, derived from references [3, 5, 6]. Note that peptides were included with small molecules (SMs) and that gene modulation, gene therapy, cell therapies (other than CAR-Ts), vaccines, IVIGs, tissue modification, and diagnostics were not included, making this number slightly different than the official US FDA count [5, 6].

In 2025, the combined FDA Center for Drug Evaluation and Research (CDER) and Center for Biologics Evaluation and Research (CBER) units [5, 6] approved 52 new (therapeutic) molecular entities (NMEs), including 31 small molecule drugs (one of which was a peptide), 10 antibody-based biologics, five gene therapies, three gene expression modulation molecules**,** and three new nonantibody biologics (one of which was a novel hyaluronidase form, shown in Table 1) [5, 6] (Fig. 1). Additionally, four vaccines and a new intravenous immunoglobulin (IVIG)–based product, which are not considered “NMEs,” received approval from the US FDA in 2025 [6]. All but one (penpulimab) of the antibodies approved by the US FDA in 2025 had previously been identified as “Antibodies to Watch in 2025” [7]. Further information on antibodies approved during the 2025 calendar year, including antibodies approved by regulatory authorities outside the USA, can be found in Crescioli *et al*. [8].

**Table 1:** Antibodies, antibody-based molecules, and antibody mimetic biologics approved by the US FDA in 2025.

Trade name (generic name)	Novel ABB	Sponsor/partner(s)	Date FDA approved	Molecular target	Approved Indication	Potential market^a^	Structure description^b^	Refs
Datroway® (datopotamab deruxtecan-dlnk)	Yes	Daiichi Sankyo/AstraZeneca	01/17/25	TROP2	AMBC; EGFR-mutated NSCLC	$5B peak	Humanized IgG1κ ADC linked by a “GGFG” tetrapeptide linker to deruxtecan; conjugation through 4 cysteinyl moieties to deruxtecan, composed of a linker and a camptothecin derivative, with an average DAR of 4 (see additional details in Table 2).	[25–27]
Anniko (安尼可 in China) (penpulimab-kcqx)	Yes	Akesobio	4/23/25^c^	PD-1	NPC	ND	Humanized IgG1κ, Fc-silenced (L234A, L235A, G236A; [Eu numbering); both C-terminal lysine residues clipped (K447Δ)	[28–30]
Imaavy® (nipocalimab-aahu)	Yes	JNJ	4/29/25	FcRn	gMGwA	$1.2B by 2030	Human IgG1λ (N297A; [Eu numbering]); nonglycosylated; both C-terminal lysine residues clipped (K447Δ)	[31–33]
Emrelis® (telisotuzumab vedotin-tllv)	Yes	Abbvie	5/14/25 ACAP	cMET	NSCLC	$0.6B by 2030	Humanized IgG1κ ADC conjugated through cysteinyl moieties to vedotin, composed of a protease-cleavable mc-val-cit-PABC linker and MMAE, with an average DAR of 3; HC: K222Δ, T223C, T225Δ, K447Δ (see additional details in Table 2)	[34–36]
Enflonsia™ (clesrovimab-cfor)	Yes	Merck	6/9/25	RSV	Prevent RSV infection	$0.84B by 2030	Human IgG1κ M252Y, S254T, T256E (“YTE”^d^) mutations to promote longer circulating half-life	[37–40]
Andembry™ (garadacimab-gxii)	Yes	CSL Behring	6/16/25	Factor XIIa	HAE	$0.42B by 2030	Human IgG4λ (S228P^e^)	[41–43]
Lynozyfic™ (linvoseltamab-gcpt)	Yes	Regeneron	7/2/25 ACAP	BCMA x CD3 bispecific TCE	R/R MM	$0.71B by 2031	Human IgG4κ (S228P^e^) heterodimeric Fc, bivalent, bispecific IgG-like antibody with CLC; HC1 (BCMA): S228P, E233P, F234V, L235A, G236Δ; HC2 (CD3ε): L112T, S228P, E233P, F234V, L235A, G236Δ, H435R, Y436F, L445P.	[44–46]
Keytruda Qlex® (pembrolizumab and berahyaluronidase alfa-pmph)	No	Genentech/Roche	09/19/25	PD-1	Several indications	$6.7B by 2030	New combination product (antibody portion is not a new NME); berahyaluronidase alfa, a new NME, is a novel variant of human hyaluronidase developed and manufactured by Alteogen Inc.^f^	[47–50]
Voyxact™ (sibeprenlimab-szsi)	Yes	Otsuka Pharma	11/25/25 ACAP	APRIL	IgAN	ND	Humanized IgG2κ	[51–53]
Lerochol® (lerodalcibep-liga)	No	LIB Therapeutics	12/12/25	PCSK9	LDL-C	ND	Not an antibody; Adnectin-HSA fusion of 77 kDa; once monthly SC injection; stable at RT for 3 months	[54–57]
Exdensur® (Depemokimab-ulaa)	Yes	GSK	12/16/25	IL-5	Asthma with polyps	$0.94B by 2031	Humanized IgG1κ M252Y, S254T, T256E “YTE”^d^ mutations to promote longer circulating half-life	[40, 58–60]
Rybrevant Faspro™ (amivantamab and hyaluronidase-lpuj)	No	JNJ	12/17/25	EGFR x cMET bispecific Ab	EGFR-mutated NSCLC	$3.9–4.0B by 2030 (both forms)	New combination product (neither antibody [IgG1-based bispecific antibody] nor hyaluronidase portion is a new NME on its own); Hyaluronidase developed and manufactured by Halozyme.	[61–62]
Yartemlea® (narsoplimab-wuug)	Yes	Omeros	12/24/25	MASP-2	TA-TMA	$0.3B peak	Human IgG4λ (S228P^e^)	[63–65]

^a^Projected market potential for 2025 FDA approved antibodies [24]

^b^Unless otherwise specified, amino acid residue numbers cited in this table are based on standard Eu antibody numbering [66]

^c^Approved in China, March 2021

^d^YTE mutation in Fc which modifies IgG binding to FcRn under acidic conditions to elongate circulating half-life [40]

^e^S228P mutation to stabilize IgG4 hinge [67, 68]

^f^Berahyaluronidase alfa produced by Alteogen [69]. Abbreviations: Ab, antibody; ABB, antibody-based biologic; ACAP, Accelerated approval by FDA; ADC, antibody-drug conjugate; AMBC, advanced or metastatic hormone receptor-positive (HR^+^), human epidermal growth factor receptor (HER)-negative (HER2^−^) breast cancer; APRIL, A Proliferation Inducing Ligand; BCMA, B-cell maturation antigen; CD, cluster of differentiation; CLC, common light chain; cMET, “proto-oncogene” mesenchymal-epithelial transition factor receptor (hepatocyte growth factor receptor); DAR, drug-to-antibody ratio; EGFR, epidermal growth factor receptor; ESCC, unresectable or metastatic esophageal squamous cell carcinoma; Fc, fragment, crystallizable; FcRn, neonatal Fc receptor; FDA, United States Food and Drug Administration; gMGwA, generalized myasthenia gravis (gMG) with anti-acetylcholine receptor (AChR) or anti-muscle-specific kinase (MuSK) antibodies; GSK, GlaxoSmithKline; HAE, hereditary angioedema; HC, heavy chain; HSA, human serum albumin; IgAN, Immunoglobulin A (IgA) nephropathy; IL, interleukin; JNJ, Johnson & Johnson; LDL-C, low-density lipoprotein-cholesterol; MASP-2, mannan-binding lectin-associated serine protease-2; mc-val-cit-PABC, maleimidocaproyl-valine-citrulline-*p*-aminobenzyl carbamate, a cathepsin-B cleavable linker/spacer; MM, multiple myeloma; MMAE, monomethyl auristatin E; ND, no data; NME, new molecular entity; NPC, recurrent or metastatic nonkeratinizing nasopharyngeal carcinoma; NSCLC, non-small cell lung cancer; PCSK9, proprotein convertase subtilisin/kexin type 9; PD-1, programmed cell death protein-1; Refs, references; R/R, relapsing/refractory; RSV, respiratory syncytial virus; RT, room (ambient) temperature; SC, subcutaneous; TA-TMA, transplant-associated thrombotic microangiopathy; TCE, T-cell engager; TROP2, trophoblast cell surface antigen-2.

Of the 169 antibody-based biologics approved by the FDA through the end of 2025, 113 are immunoglobulin G (IgG)–based antibodies, 16 are Fc fusions or Fc-based proteins, 15 are bispecific antibodies (of which 10 are T-cell engagers), 14 are antibody–drug conjugates (ADCs), 6 are antibody fragments, 2 are antibody mixtures, 2 are radioimmunoconjugates (RICs), and 1 is an antibody–toxin fusion protein [3]. As noted in last year’s summary [9], this count excludes the emergency use authorizations (EUAs) for four anti-severe acute respiratory syndrome (SARS)–coronavirus-2 (CoV-2) antibodies/antibody mixtures [10].

Since 1997, when antibody-based biologics began truly taking off as therapeutic options, the annual FDA approval rate has been about six new antibody-based biologics approved each year [3]. Over the past 12 years, however, from 2014 to 2025, 125 new antibody-based drugs, which includes IgGs, bispecific antibodies, ADCs, antibody fragments, and Fc fusion proteins, were approved by the US FDA [3]. This amounts to a mean and median of 10.4 and 10 new antibody-like molecules approved per year, respectively, during that period (Fig. 1). Thus, the 10 new antibodies approved by the FDA in 2025 is entirely consistent with those mean and median numbers. From 1997 and 2013, the period preceding this recent 12-year span, the FDA had approved an average of only 2.35 new antibodies each year [11]. Thus, the vastly increased number of antibodies approved by the FDA annually in the past dozen years represents a “new norm,” as well as the maturation of the field.

**Figure 2:**
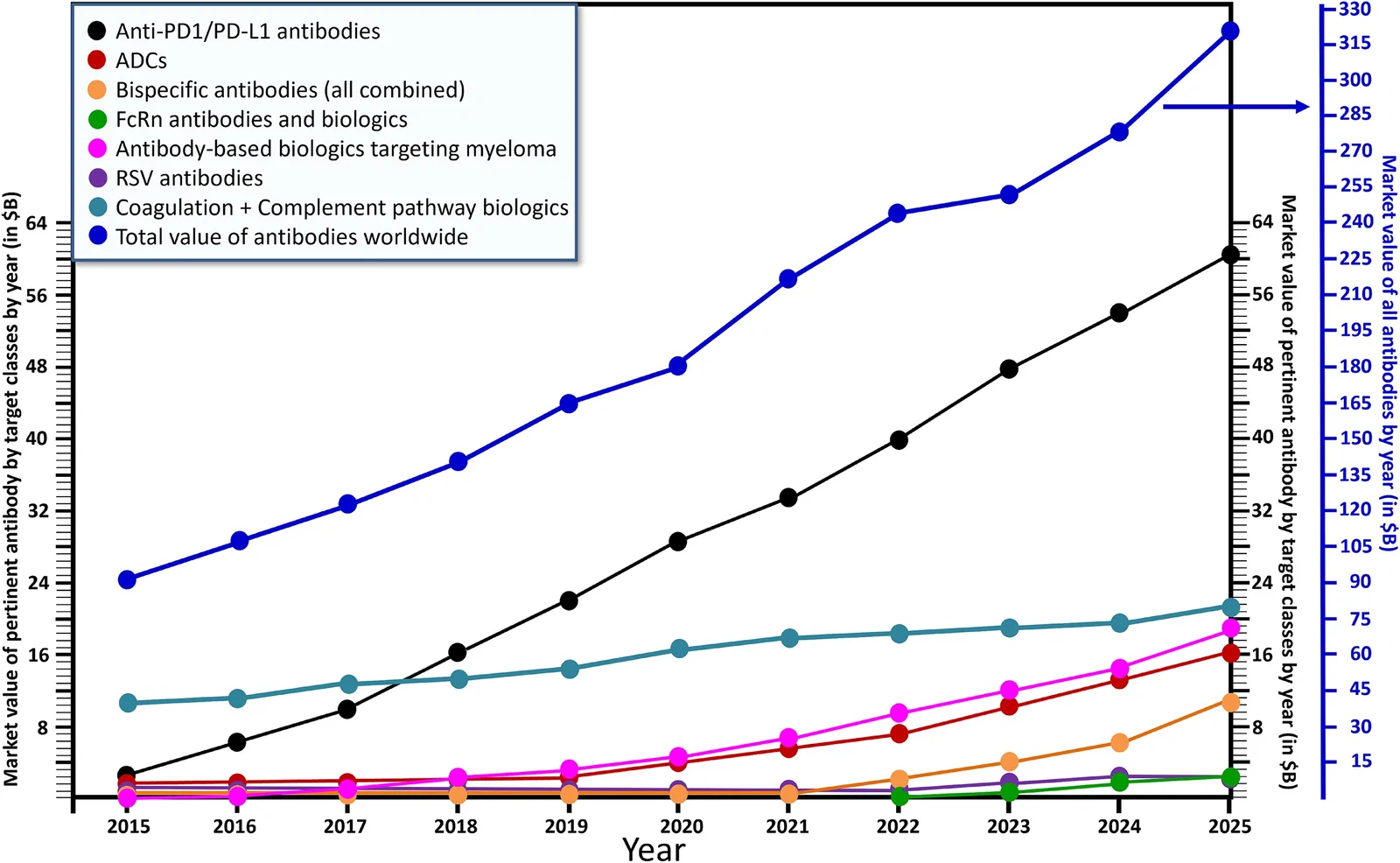
The historical value of markets from 2015 to 2025 for those markets represented in the new antibodies approved in 2025. These include anti-programmed cell death protein-1 (PD-1)/programmed cell death protein ligand-1 (PD-L1) mAbs, bispecific antibodies, antibody-drug conjugates (ADCs), biologics targeting the neonatal Fc receptor (FcRn), antibodies targeting respiratory syncytial virus (RSV), antibody-based biologics targeting multiple myeloma, and antibodies and biologics (all combined) targeting the Complement and Coagulation pathways (combined). The total market value of antibody-like biologics worldwide is also included (furthest right-side numbers). These data are derived from references [4, 12–22].

During this recent 12-year period (2014–25), the global market value of antibodies surged from $64.6 billion (USD) in 2014 to ~$321 billion (USD) in 2025 [4, 12–22] (Fig. 2), nearly quintupling in value over that period. This reflects an almost linear average increase in sales of ~$18 billion (USD) per year (Fig. 2). The projection for the antibody market size eight years from now, in 2034, is $1,058 billion, with an average compound annual growth rate (CAGR) of ~15% over the period of 2025–34 [23]. It is important to note that antibody sales figures are a multi-year lagging indicator and cannot be used for precise future predictions.

## Potential valuations of the newcomers

The 2025 class of FDA-approved antibodies should contribute to the increased market size for antibodies as documented in Table 1, although perhaps not as strongly as the 2024 class of newly approved antibodies [9]. The potentially most valuable of the 2025 approved antibodies is the anti-trophoblast cell surface antigen-2 (TROP2) ADC, Datroway® (datopotamab deruxtecan), which is projected to have peak sales of $5 billion [24]. The likely next most valuable newcomer, with a projected value in 2030 of $1.2 billion, is Imaavy® (nipocalimab), which is entering the highly competitive neonatal Fc receptor (FcRn) target market [24]. The rest of the newly approved antibody NMEs are projected to peak under the $1 billion mark (Table 1). The two new hyaluronidase-containing subcutaneous formulations, Keytruda QLEX® (pembrolizumab-berahyaluronidase coformulation) and Rybrevant Faspro® (amivantamab–hyaluronidase coformulation), will provide significant market expansion, and especially for Keytruda, market extension postpatent expiration in 2028, for each of those franchises, as well as providing patients with significantly more user-friendly anticancer antibody treatment paradigms.

## Overview of 2025 FDA-approved antibody-based biologics

### Targets and indications

The 10 novel antibody-like biologics approved by the US FDA in 2025 include molecules targeting both well-known pathways for which other molecules have been approved and antibodies targeting antigens for which no previous antibodies have been approved. Seven of the 10 newly approved antibody-based biologics are against target proteins for which the FDA has previously approved biologics (Table 1). These include: (i) penpulimab, the eighth FDA-approved antiprogrammed cell death receptor-1 (PD-1) mAb [30]; (ii) linvoseltamab, the fourth antibody approved by the FDA to target B-cell maturation antigen (BCMA) and the third to target both BCMA and CD3ε as a T-cell engager (TCE) [46]; (iii) nipocalimab, the third FDA-approved biologic FcRn antagonist [33]; (iv) clesrovimab, the third FDA-approved mAb targeting respiratory syncytial virus (RSV) to prevent infant respiratory infections [39]; (v) depemokimab, the third antibody to target the IL-5 pathway [60]; (vi) datopotamab deruxtecan, the second mAb and ADC to target TROP2 [27]; and (vii) telisotuzumab vedotin, the second FDA-approved antibody, albeit the first ADC, to target mesenchymal–epithelial transition (receptor) cMET [36].

The remaining three newly FDA-approved novel antibodies are directed toward novel targets for which antibodies have not previously been approved by the FDA. These include: (i) garadacimab, which targets Factor XIIa in the coagulation cascade, for treatment of hereditary angioedema (HAE) [43]; (ii) sibeprenlimab, the first FDA-approved antibody to target APRIL (“a proliferation-inducing ligand”) specifically, for treatment of IgA-mediated nephropathy (IgAN) [53]; and (iii) narsoplimab, which targets the mannan-binding lectin-associated serine protease-2 (MASP-2), a complement lectin pathway initiation protein, for treatment of hematopoietic stem cell transplant–associated thrombotic microangiopathy (TA-TMA) [65].

Additionally, two new antibody-like biologics combination products, Keytruda QLEX® [49, 50] and Rybrevant Faspro® [62], were approved in 2025 (Table 1). While the antibody-like biologics portions of Keytruda QLEX® and Rebryvant Faspro® were not new, the combination with a hyaluronidase with each to promote fast and efficient subcutaneous (SC) dosing is new. These new formulations allow for SC dosing of high concentrations and volumes of antibody in substantially faster regimens than experienced with intravenous (IV) dosing. As noted previously [9], the hyaluronidase functions to degrade the gel-like hyaluronic acid in the subcutaneous layer, which increases the local volume available for drug to enter.

Finally, as shown in Table 1, a final non-antibody biologic product, Lerochol® (lerodalcibep-liga) [56], which was approved by the FDA in 2025, is being highlighted here because it is an antibody-mimetic scaffold (“adnectin”) that functions essentially like an antibody (e.g. binds to a specific epitope on a target protein, proprotein convertase subtilisin/kexin type 9 [PCSK9]), to block its natural function [57]. Lerodalcibep joins ecallantide (Kalbitor®), which was approved in 2009 for treatment of HAE [70], as the only antibody-like mimetic scaffolds to be FDA-approved thus far.

### Structural features of the newly approved antibody-based biologics

**Figure 3:**
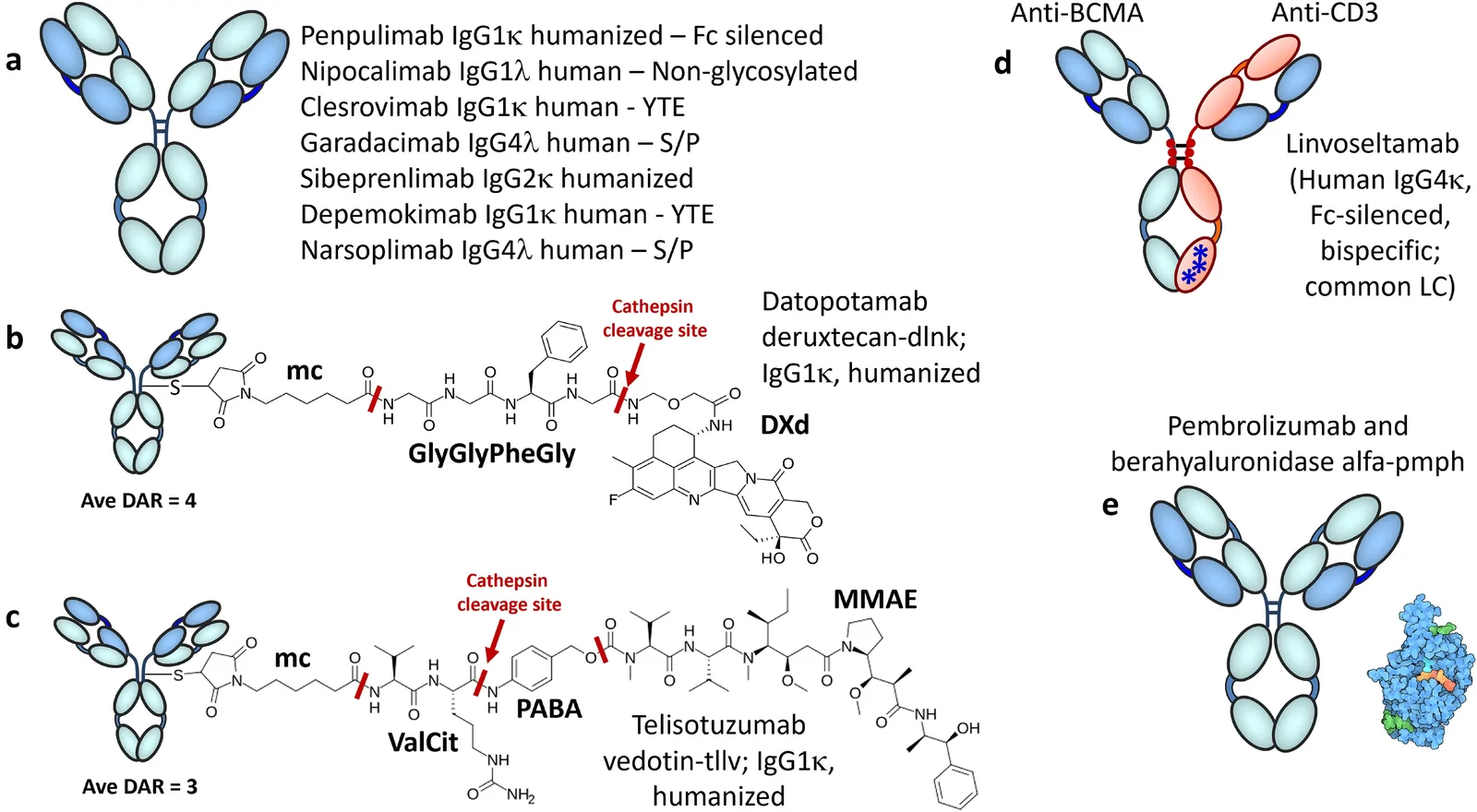
Cartoons showing the generalized structures of the 10 new antibody-based biologics and one new combination product containing a novel protein structure approved by the US FDA in 2025. (a) Seven of the antibodies are IgGs, one of which has been engineered to significantly reduce binding to FcγRs and C1q (i.e. “silenced”), one engineered to reduce FcγR binding via non-*N*-glycosylation at N297, two engineered by incorporating the “YTE” mutations to significantly increase circulating half-life; and two IgG4s engineered to stabilize the hinge (see Table 1). (b) Datopotamab deruxtecan is a new humanized IgG1κ ADC to which the cytotoxic modified natural product, Deruxtecan (exatecan derivative; DXd) is linked to cysteine residues via a protease cleavable, maleimidocaproyl (mc)-conjugated tetrapeptide (GlyGlyPheGly) linker with an average DAR of 4. (c) Telisotuzumab vedotin is a new humanized IgG1κ ADC to which the cytotoxic modified natural product, monomethyl auristatin E (MMAE), is linked to cysteine residues via cathepsin-cleavable maleimidocaproyl valine-citrulline-*p*-aminobenzyl carbamate linker/spacer with an average DAR of 3. (d) Linvoseltamab is a bivalent, heterodimeric-Fc human IgG4κ common light-chain format bispecific TCE antibody in which one Fab arm binds BCMA and the other binds CD3ε. The antibody is mutated to be Fc-silenced, and one Fc half is mutated to alter its Protein A–binding characteristics to aid in purification. (e) Pembrolizumab and berahyaluronidase alfa-pmph is a new combination product with a novel form of hyaluronidase for subcutaneous delivery of Keytruda®.

Of the 10 novel antibody-based proteins approved by the US FDA in 2025, none was a canonical “naked” IgG1 antibody with wild-type Fc. Since 2004, this phenomenon has also occurred in three other years, 2008, 2019, and 2022, noting that in the past 20 years or so, the field has increasingly accepted noncanonical IgGs. In 2025, six approved antibodies were based on the IgG1 isotype, but one was Fc-silenced (penpulimab), one was nonglycosylated (nipocalimab) to make it Fc-muted, two contained the half-life extending YTE mutations (clesrovimab, depemokimab), and two were wild-type humanized IgG1-based ADCs (datopotamab deruxtecan, telisotuzumab vedotin) (Table 1, Fig. 3). Of the other IgGs, one was an IgG2 (sibeprenlimab), and three were of the IgG4 isotype (garadacimab, narsoplimab, and the bispecific IgG, linvoseltamab). As is practically universal these days, the IgG4 antibodies approved in 2025 all possess the S228P mutation (Eu numbering [66]) to stabilize the hinge, preventing half-antibody formation [67, 68]. The variable regions of six of the newly approved antibodies were derived from human genes, and four were humanized from mouse hybridomas and then humanized (Table 1).

### Antibody–drug conjugates

As noted above, two of the antibodies approved by the FDA in 2025 were ADCs (Tables 1 and 2), which brings the total number of ADCs approved by the FDA to 14 (Table 2). The first ADC approved by the FDA, Mylotarg®, was approved in 2000 [71], making 2025 the 25th year anniversary of the first marketing approval for ADCs [72]. The technology for generating ADCs has come a long way since 2000, with marked improvements in all four critical aspects of an ADC: (i) improved antibodies, targeting better characterized targets [73]; (ii) better conjugation site management, with many newer ADCs site-specifically conjugated and sporting well-characterized drug-to-antibody ratios (DARs) [74, 75]; (iii) improved linker technologies that provide greater stability while the ADC is in circulation, followed by specific payload disbursement when the ADC is internalized by target cells [74, 76]; and (iv) improved toxic payloads [77]. With these improvements in ADC design and structure, it is important to note that half of the ADCs approved by the FDA have been approved just since 2020 (Table 2). This is also reflected in the market data shown in Fig. 2, whereby the ADC market as a whole has increased four-fold from ~$4 billion in 2020 to $16 billion in 2025 (Fig. 2).

**Table 2:** Fourteen antibody–drug conjugates (ADCs) and one antibody–toxin conjugate approved by the US FDA.

Name of ADC	Sponsor	Date FDA approved	Target	Primary indica-tion	Payload	Linker/cleavage	DAR (ave)	Bystander effect	Antibody	Refs
Mylotarg® (gemtuzumab ozogamicin)	Wyeth (Pfizer)	05/17/00; Discontinued 6/21/10; Re-approved 10/1/17	CD33	AML	CLCM	4-(4-Acetyl-phenoxy) butanoic acid; pH sensitive	2–3	Yes	Humanized IgG4κ	[71, 79]
Adcetris® (brentuximab vedotin, SGN-35; cAC-10)	Seattle Genetics (Pfizer)/Takeda	8/19/11	CD30	HL	MMAE	mc-ValCitPABC; lysosomal protease degradation	4	Yes	Chimeric IgG1κ	[80–82]
Kadcyla® (trastuzumab emtansine; aka T-DM1)	Roche/Genentech	2/23/13	HER2	MBC	DM1	MCC; noncleavable linker	3.5	No	Humanized IgG1κ	[83–85]
Besponsa® (inotuzumab ozogamicin; CMC-544)	Pfizer/UCB	8/17/17	CD22	B-ALL	CLCM	4-(4-Acetyl-phenoxy) butanoic acid; pH sensitive	~6	Yes	Humanized IgG4κ	[86–87]
Polivy® (polatuzumab vedotin-piiq; RG7596, DCDS4501A)	Genentech/Chugai/Seattle Genetics (Pfizer)	6/10/19	CD79b	DLBCL	MMAE	mc-ValCitPABC; lysosomal protease degradation	3.5	Yes	Humanized IgG1κ	[81, 88, 89]
Padcev® (enfortumab vedotin-ejfv; ASG-22ME)	Astellas/Seattle Genetics (Pfizer)	12/18/19	Nectin-4	MUC	MMAE	mc-ValCitPABC; lysosomal protease degradation	3.8	Yes	Human IgG1κ	[81, 90, 91]
Enhertu® ([fam]-trastuzumab deruxtecan-nxki; DS-8201a)	Daiichi-Sankyo/AstraZeneca	12/20/19	Her2	MBC	DXd	Site-specific conjugation; MC-GGFG-AM; lysosomal protease degradation	8	Yes	Humanized IgG1κ	[92–93]
Trodelvy® (sacituzumab govitecan; IMMU-132; hRS7-SN38)	Immunomedics/Seattle Genetics (Pfizer)	04/22/20	TROP2	TNBC, PANC	SN-38	CL2A linker; pH sensitive; cleaved chemically in acidic lysosome	7.6	Yes	Humanized IgG1κ	[94–95]
Blenrep® (belantamab mafodotin-blmf; GSK2857916)	GSK/Seattle Genetics (Pfizer)	08/05/20; Withdrawn 11/22/22; Re-approved 10/26/25	BCMA	R/R MM	MMAF	Maleimidocaproyl (mc) linker; Protease-resistant, noncleavable	4	No	Humanized IgG1κ; afucosylated	[96–98]
Zynlonta® (loncastuximab tesirine-lpyl; ADCT-402)	ADC Therapeutics SA	4/23/21	CD19	DLBCL	PDB-D	Protease-cleavable (ValAla-PABC) dipeptide	3.5	Yes	Humanized IgG1κ	[99–101]
Tivdak® (tisotumab vedotin; HuMax®-TF-ADC)	Genmab/Seattle Genetics (Pfizer)	9/20/21	TF	Cervical cancer	MMAE	mc-ValCitPABC; lysosomal protease degradation	4.0	Yes	Human IgG1κ	[81, 102, 103]
Elahere® (mirvetuximab soravtansine-gynx; IMGN853)	ImmunoGen (Abbvie)	11/14/22	FRα	Ovarian cancer	DM4	Sulfo-SPDB; cleaved in lysosome via reductive potential (e.g. GSH)	3.4	Yes	Humanized IgG1κ	[104– 105]
Datroway® (datopotamab deruxtecan; DS-1062a)	Daiichi Sankyo/AstraZeneca	1/17/25	TROP2	NSCLC	DXd	Protease-cleavable tetrapeptide (GGFG)-based linker; cysteine-conjugated	4–4.2	Yes	Humanized IgG1κ	[25, 26]
Emrelis® (telisotuzumab vedotin-tllv)	Abbvie	5/14/25 Accelerated approval	cMET	NSCLC	MMAE	mc-ValCitPABC; lysosomal protease degradation; cysteine-conjugated	3.1	Yes	Humanized IgG1κ	[34, 35, 81]

Abbreviations: ADC, antibody–drug conjugate; aka, also known as; AML, acute myeloid leukemia; B-ALL, B-cell acute lymphoblastic leukemia; BCMA, B-cell maturation antigen; CD, cluster of differentiation; CL2A, pH-sensitive, cleavable polyethylene glycol-8- and triazole-containing *p*-aminobenzyl carbamate-peptide-mc linker; CLCM, Calicheamicin; cMet, receptor tyrosine kinase mesenchymal–epithelial transition factor; DAR, drug-to-antibody ratio; DLBCL, diffuse large B-cell lymphoma; DM1, mertansine, a maytansinoid derivative; DM4, ravtansine, a maytansinoid derivative; DXd, Deruxtecan (exatecan derivative); EGFR, epidermal growth factor receptor; FDA, US Food and Drug Administration; FRα, folate receptor-alpha; GSH, glutathione; GSK, GlaxoSmithKline; HER2, human epidermal growth factor receptor-2; HL, Hodgkin lymphoma; HLA-A2, human leukocyte antigen-A2; MBC, metastatic breast cancer; mc, maleimidocaproyl moiety; MCC, 4-[*N*-maleimido-methyl] Cyclohexane-1-carboxylate linker; mc-GGFG-AM, maleimidocaproyl-glycyl-glycyl-phenylalanyl-glycine-amino methyl (linker to drug); mc-ValCitPABC, cathepsin-cleavable maleimidocaproyl valine-citrulline-*p*-aminobenzyl carbamate linker/spacer; MM, multiple myeloma; MMAE, monomethyl auristatin E; MMAF, monomethyl auristatin-F; MUC, metastatic urothelial cancer; NSCLC, non-small-cell lung cancer; PANC, pancreatic cancer; PDB-D, pyrrolobenzodiazepine dimer; Refs, references; R/R, relapsing/refractory; SN-38, 7-ethyl-10-hydroxycamptothecin, a highly (100–1000-fold more potent) active metabolite of irinotecan (CPT-11); Sulfo-SPDB, (1-(2,5-dioxopyrrolidin-1-yl)-oxo-4-(pyridin-2-yldisulfanyl) butane-2-sulfonic acid); TF, tissue factor; TNBC, triple-negative breast cancer; TROP2, transmembrane glycoprotein encoded by the Tacstd2 gene; ValAla-PABC, valyl-alanine-*p*-aminobenzyl carbamate.

The shift toward increased reliance on ADCs to treat solid tumors is rapidly gaining momentum. There are at least 33 unique ADCs currently in Phase 3 clinical trials as documented in Clinicaltrials.gov, including nine targeting HER2, and four apiece targeting Claudin 18.2 and TROP2, and three targeting B7H3 [3]. Within the next 4–5 years, one would expect that a significant number of these should gain approval by regulators and be added to the 14 ADCs already FDA-approved. Finally, according to BEACON [78], 458 ADC candidates have entered clinical trials over the past 10 years, with 130 new ADC candidates entering the clinic in 2025 alone [78]. Thus, it appears that the development of ADCs as potent antitumor drugs has finally hit its stride, with heightened expectations for continued expansion in terms of use and clinical success.

The ADCs approved in 2025 by the FDA represent two very different forms of ADCs, with different DARs, linkers, and toxic payloads (Table 2, last two entries). The first of these is Datroway® (datopotamab deruxtecan), targeting TROP2 with a Dxd payload linked to the antibody via a protease cleavable tetrapeptide linker, while the second is Emrelis® (telisotuzumab vedotin-tllv), which targets cMET, a proto-oncogene receptor tyrosine kinase (note: the “c” of cMET designates it as a proto-oncogene) with an MMAE payload conjugated to the antibody via a cathepsin-cleavable maleimidocaproyl valine-citrulline-*p*-aminobenzyl carbamate linker/spacer.

### Half-life extended antibodies

An analysis of 45 FDA-approved therapeutic IgG-based antibodies that bind soluble targets (to minimize the effects of target-mediated clearance on the dataset) reveals that they have a median and mean circulating half-life in humans of 18 and 19 days, respectively, based on data from the package inserts [3]. Some, such as fremanezumab [106], ustekinumab [107], eptinezumab [108], and emicizumab [109], each of which possesses a mean circulating terminal half-life in humans in the range of 27–31 days [3], have long average circulating half-lives, while at the other end of the spectrum, concizumab has a circulating terminal half-life of only 1 day [110]. In most cases, the longer an antibody persists in circulation, i.e. available for binding its target, the better. Longer half-life increases the area under the curve (AUC), resulting in greater total exposure of the drug to its target. Moreover, longer circulating half-life directly translates into less frequent dosing. Thus, several groups have generated mutations in either the IgG Fc [e.g., 40, 111–113] or Fv [e.g., 114, 115] regions, or both [116], to improve the circulating half-life of IgG-like antibodies.

Table 3 provides an overview of some of the approved and late clinical-stage antibodies that have been modified to improve circulating half-life. The most notable of the Fc mutations are “YTE” (M252Y, S254T, T256E [40], Eu numbering [66]) and “LS” (M428L, N434S [111], Eu numbering [66]), which were invented by scientists at MedImmune (AstraZeneca) [40] and Xencor [111], respectively. The YTE mutation technology patent expired 17 January 2022 [117], allowing investigators everywhere to utilize this important tool to improve antibody half-life. This is so significant that at least two companies, Spyre Therapeutics [118] and Apogee Therapeutics [119], have recently sprung up that focus on using the YTE mutation to improve the circulating half-life of existing antibodies (to make “me-betters”), as well as novel half-life extended candidates.

**Table 3:** Examples of approved or late-stage half-life extended IgG antibodies.

US trade name (generic name)	Company or sponsor	Approval status	Molecular target	Major Indication	Half-life (days)	Clearance and dosing frequency	Protein format and notes ^ a^	Refs
Ultomiris® (ravulizumab-cwvz)	Alexion Pharma (now AstraZeneca)	FDA approved 12/21/18	C5	PNH	50 d (ave)	CL, 0.022 L/d; Dosing, Q8W	Humanized IgG2/4 k; Xtend LS plus V-chain mutations	[116, 124]
Xevudy® (sotrovimab)	GSK/Vir Biologics	FDA EUA granted 5/26/21; EUA revoked 4/5/22	SARS-CoV-2 spike protein	COVID-19	80–89 d	CL, 0.067 L/d; Dosing, Q8W	Human IgG1k; Xtend LS mutations	[10, 125, 126]
Evusheld® (AZD7442) (Tixagevimab IgG1k + Cilgavimab IgG1k)	AstraZeneca	FDA EUA granted 12/8/21; EUA revoked 1/26/23	SARS-CoV-2 spike protein	Prevent SARS-CoV2 infection	TIX: 90d; CIL: 80–91 d	TIX CL, 0.062 L/d; CIL CL, 0.074 L/d; Dosing, Q6M	Fixed dose combination of two human IgG1ks, both with YTE mutations	[10, 121, 127]
Beyfortus® (nirsevimab-alip)	AstraZeneca	FDA approved 7/17/23	RSV	Prevent RSV infection	71 d	CL, 0.0034 L/d; Dosing, Q5M	Human IgG1; YTE mutations	[128, 129]
Piasky® (crovalimab-akkz)	Roche	FDA approved 6/20/24	C5	PNH	53 d	CL, 0.08 L/d; Dosing, Q1M	Human IgG1; SMART-Ig technology	[130, 131]
Enflonsia® (Clesrovimab-cfor)	Merck	FDA approved 6/9/25	RSV	Prevent RSV infection	44 d	CL, 0.020 L/d; Dosing, Q5M	Human IgG1; YTE mutations	[37, 38, 132]
Exdensur® (Depemokimab-ulaa)	GSK	FDA approved 12/16/25	IL-5	Asthma with polyps	48 d	CL, 0.092 L/d; Dosing, Q6M	Humanized IgG IgG1k; YTE mutations (NCT06961214)	[58, 59, 133, 134]
Barzolvolimab CDX-0159	Celldex	Phase 3	CD117 (aka c-kit)	CIU	32–44 d	CL, 0.175 L/d; Dosing, tbd	Humanized IgG; YTE mutations (NCT06455202*)	[135, 136]
Recaticimab	Jiangsu Hengrui Pharmaceuticals	Phase 3	PCSK9	LDL-C	19–27 d	CL, 0.3–0.4 L/d Q4W, Q8W, Q12W dosing projected	Human IgG1; YTE mutations (NCT07119918)	[137, 138]
CIS43LS	Liverpool School of Tropical Med	Phase 3	P falciparum CSP-1	Malaria	56 d	CL, 0.044 L/d Dosing, tbd	Human IgG; Xtend LS mutations (NCT07082205)	[139, 140]
Suvratoxumab (MEDI-4893, AR-320)	Aridis, from MedImmune/AZ	Phase 3 terminated	*S. aureus* alpha toxin	*S. aureus* alpha toxin	80–112 d	CL, 0.042 L/d; Dosing, tbd	Human IgG1k; YTE mutations; (NCT05331885 terminated 7/6/24)	[141, 142]
Zumilokibart APG777	Apogee Therapeutics	Phase 2	IL-13	AD	75–77 d	CL, 0.094 L/d 3.6–3.9 mL/h	Humanized IgG1 LALA; YTE mutations (NCT07003425)	[143, 144]
VRC01LS	NIH	Phase 2	HIV-1 gp120	AIDS	71 +/− 18 d	CL, 0.036 L/d	Human IgG1λ; Xtend LS mutations (NCT07128550)	[145, 146]
VIR2482	Vir Biotech-nology Corp	Phase 2	Influenza A virus	influenza	56.7–70.6 d	CL, 0.13–0.15 L/d; Q3M to Q6M dosing projected	Human IgG1; Xtend LS mutations (NCT05567783)	[147, 148]
Tobevibart (VIR-3434)	Vir Biotech-nology Corp	Phase 2	HBV surface antigen	HBV infection	19–21 d	CL, 0.4 L/d Q2W to Q4W projected	Human IgG1λ; Xtend LS mutations; (NCT07128550)	[149, 150]
Teropavimab (3BNC117-LS) and Zinlirvimab (10-1074-LS)	ACTG	Phase 2	CD4 HIV binding site	HIV/AIDS	3BNC-LS, 66 d 1074-LS, 80 d	Q20W dosing in NCT05612178	Fixed dose combination of two human IgGs; Xtend LS mutations; (NCT06205602)	[151, 152]
SPY002	Spyre Therapeutics	Phase 2	TL1A	UC	Ca. 75 d	Q3M to Q6M dosing projected	IgG; YTE mutations (NCT07012395)	[153, 154]
SPY003	Spyre Therapeutics	Phase 2	IL-23p19	IBD	Ca. 85 d	Q3M to Q6M dosing projected	IgG; YTE mutations (NCT07012395)	[154, 155]
Xmab942	Gale Therapeutics (Xencor)	Phase 1/2	TL1A	UC	71 d (est’d)	Q12W dosing projected	IgG; Xtend LS mutations; (NCT06619990)	[156, 157]
Pemivibart	Certera	Phase 1	SARS-CoV-2 spike protein	COVID-19	49 d	0.086 L/d; Q90D projected	Human IgG1λ; MNLA mutations	[158]
22 antibodies listed in this table	NA	All clinical phases	All “soluble” targets	NA	Mean: 63 d Median: 71 d	Mean: 0.10 L/d Median: 0.074 L/d N = 17	HLE mutations used: YTE, 11; LS, 9; SMART-Ig, 1; MNLA, 1,	NA

Abbreviations: ACTG, Advancing Clinical Therapeutics Globally for HIV/AIDS and Other Infections; AD, atopic dermatitis; AIDS, acquired immunodeficiency syndrome; aka, also known as; Ave, average; CD, cluster of differentiation; CIL, Cilgavimab; CIU, chronic idiopathic urticaria; CL, clearance in L/d; COVID, Corona virus disease; EUA, Emergency Use Authorization; FDA, US Food and Drug Administration; GSK, GlaxoSmithKline; HBV, hepatitis B virus; HIV, human immunodeficiency virus; IBD, inflammatory bowel disease; IgG, immunoglobulin G; IL, interleukin; L/d, liters per day; LDL-C, low-density lipoprotein-cholesterol; NA, not applicable; *P. falciparum, Plasmodium falciparum*; PNH, paroxysmal nocturnal hemoglobinuria; Q, “Quaque” (Latin; each, every), as in Q4W (every 4 weeks), Q2M, every 2 months, etc.; Refs, references; RSV, respiratory syncytial virus; SARS-CoV-2, severe acute respiratory syndrome coronavirus-2; *S. aureus, Staphylococcus aureus*; TIX, Tixagevimab; TL1A, tumor necrosis factor–like ligand A (TNFSF15); UC, ulcerative colitis; V-chain, variable chain.

^a^All positions are numbered according to Eu numbering [66]. Mutants mentioned include YTE mutations, M252Y/S254T/T256E to extend circulating half-life [40]; Xtend LS mutations, M428L/N434S to extend circulating half-life [111]; MNLA, M435L/N441A to extend circulating half-life [159]; LALA mutation, L234A/L235A to reduce but not completely eliminate FcγR interactions (all modifications are given based on Eu numbering [160]).

These Fc mutations enhance the binding of the IgG to the FcRn at acidic pH with little or no binding at neutral pH, which results in increased recycling of the antibody and a longer time in circulation [120]. Of the antibodies approved by the US FDA in 2025, two of them (clesrovimab and depemokimab) possess the YTE mutation in their Fc regions to significantly improve their half-life over canonical IgGs. To date, including the two new half-life extended (HLE) antibodies approved in 2025, a total of four HLE-IgGs have been approved for marketing and another three, all of them anti-SARS-CoV2 antibodies, were granted EUAs (Table 3). Two of those, HLE-tixagevimab and HLE-cilgavimab, were dosed together in a formulation named Evusheld® [121]. Of these seven HLE-IgGs noted in Table 3 as FDA-approved or EUA-authorized, it is noteworthy that five are antiviral antibodies. The mean and median values for the 22 HLE-IgGs noted in Table 3 are 63 d (where ranges were given, the midpoint was used) and 71 d, respectively, with a range of 19–21 days on the low end to a high end of 80–122 days (Table 3). As a group, these represent circulating half-life improvements over the “typical” IgGs in the range of about three-to-four-fold. One note of caution in interpreting these data is that they represent different antibody data sets, with multiple factors including IgG sequence and structure, antibody charge (e.g. pI), target concentration, biodistribution, dosing levels, and other factors contributing to circulating half-life [122, 123].

The following sections describe each of the antibody newcomers approved by the FDA in 2025. The two ADCs will be reviewed first, followed by the two half-life extended antibodies, the one new bispecific TCE antibody, and the two very novel antibody approaches targeting the complex coagulation and complement cascades. These will be followed by the two antibodies neutralizing targets associated with immune cells, the anti-FcRn antibody, and then the two antibodies coformulated with hyaluronidase for large volume subcutaneous delivery. The final entry will be a short discussion of the antibody-like scaffold targeting PCSK9, essentially competing in an antibody-dominated market.

## Datroway (datopotamab deruxtecan-dlnk) - anti-TROP2 ADC

### Trophoblast cell surface antigen 2

Datroway® (datopotamab deruxtecan-dlnk; aka Dato-DXd) recognizes TROP2, also known as epithelial glycoprotein-1 (EGP-1) and tumor-associated calcium signal transducer-2 (TACSTD2) [161–163]. TROP2 is a single pass transmembrane glycoprotein that can dimerize or form stable tetramers that function to regulate calcium signaling involved in cell adhesion, proliferation, survival, and differentiation [161–163]. TROP2 has a large extracellular domain, a helical transmembrane region, and an intracellular region, or cytoplasmic tail, that is phosphorylated by protein kinase C (PKC) to activate RAF kinase and NF-κB [161, 162]. Encoded by the *TACSTD2* gene, TROP2 is expressed at low to moderate levels on epithelial cells and is important in normal tissue regeneration, proliferation, and differentiation [162–164]. The highest expression in normal tissues is in urothelium, endometrium, placental tissue, and prostate basal cells [163]. TROP2 is overexpressed in many epithelial-derived (e.g. breast, gastric, lung, colorectal, pancreatic) cancers, and its overexpression is often associated with poor clinical outcomes [163, 164].

Because of its overexpression in certain cancerous tissues and relatively low expression in normal tissues, TROP2 has become an important target for ADCs like Trodelvy® (sacituzumab govitecan), which binds TROP2 and delivers the cytotoxic agent, SN-38 (highly potent, active metabolite of camptothecin) [94, 95, 165], to tumor cells while largely sparing normal tissue. Sacituzumab govitecan, which was approved by the FDA in 2020, has been indicated for treatment of unresectable triple-negative breast cancer (mTNBC) and hormone receptor-positive (HR^+^), HER2-negative breast cancer [95, 165].

### Hormone receptor–positive, human epidermal growth factor receptor 2-negative breast cancer

Breast cancer (BC) is the most common form of cancer in women, with 2.3 million cases and 670 000 deaths in 2022 [166]. BC can either be noninvasive, such as ductal carcinoma *in situ* (DCIS) and lobular carcinoma *in situ* (LCIS), or invasive, characterized by stromal infiltration and dissemination, including the subtypes invasive ductal carcinoma (IDC), invasive lobular carcinoma (ILC), and inflammatory breast cancer (IBC), as well as some less common carcinomas [166].

Additionally, BC can be characterized into four major groupings by their molecular phenotype, including the presence or absence of the hormone receptors (HRs), estrogen receptor (ER) and progesterone receptor (PR), as well as HER2 [166]. Breast tumors that express both hormone receptors, ER and PR, but not HER2 (e.g. HR^+^ [i.e. both ER^+^ and PR^+^]/HER2^−^) are sometimes referred to as “Luminal A” tumors; Luminal B tumors express ER and HER2, may or may not express PR, and are often more proliferative; HER2-positive tumors are typically ER and PR-negative; and triple-negative breast cancer is characterized by not expressing ER, PR, or HERs (ER^−^, PR^−^, HER2^−^) BC (TNBC) [166].

For HER2^+^ BC, there are now several antibody-based treatment options, including the anti-HER2 antibodies such as Herceptin® (trastuzumab), Perjeta® (pertuzumab), or a combination of those two anti-HER2 antibodies, the high antibody-dependent cellular cytotoxicity (ADCC) mAb, Margenza® (margetuximab), the bispecific, biparatopic anti-HER2, Ziihera® (zanidatamab), the HER2/HER3-bispecific antibody, Bizengri® (zenocutuzumab), and the two anti-HER2 ADCs, Kadcyla® (trastuzumab emtansine) and Enhertu® ([fam]-trastuzumab deruxtecan) [9, 167].

Of the 300 000-plus cases of breast cancer that are diagnosed annually in the USA, ~70% fall into the category of HR-positive, HER2-negative breast cancer (measured as HER2 score of IHC 0, IHC 1^+^, or IHC 2^+^/ISH^−^) [168]. Tamoxifen is often prescribed for early-stage HR^+^/HER2^−^ breast cancer as a hormone receptor antagonist [169]. Following tamoxifen therapy, which has limitations, historical treatment of HR^+^/HER2^−^ BC has typically included surgery, chemotherapy, or radiotherapy. As noted above, Trodelvy® (sacituzumab govitecan) has also been approved for treatment of both triple-negative and HR^+^/HER2^−^ BC [98, 165].

### Non-small-cell lung cancer

Lung cancer is the leading cause of death in cancer, with ~20% of all cancer deaths attributed to lung cancer. In 2022, there were ~2.48 million new cases of lung cancer worldwide, with over a million new cases in China and another quarter million in the USA [170]. Non-small-cell lung cancer (NSCLC) comprises ~81% of all lung cancer cases, with small-cell lung cancer (SCLC, ca. 14%) as the second most prevalent form [171]. The two major forms of NSCLC are adenocarcinoma (NSCLC-AC), comprising ~52% of all NSCLC cases, and squamous cell carcinoma (NSCLC-SCC), which makes up ~35% of cases [171]. Additionally, large cell carcinoma, which is a relatively minor, but aggressive, form of lung cancer affects a small population of NSCLC patients.

NSCLC-AC typically starts in the mucous-producing glandular cells at the periphery of the lung and, in general, grows more slowly than other lung cancers. NSCLC-AC is largely associated with driver mutations such as those in KRAS, and the genes encoding epidermal growth factor receptor (EGFR), cMET, anaplastic lymphoma kinase (ALK), and other proteins [172]. Currently, many NSCLC-AC therapies target cancers with specific mutations. NSCLC-SCC, on the other hand, originates in the squamous cells in the central region of the lung and is associated with heavy smoking [173]. NSCLC-SCC, which is not typically associated with driver mutations, is more aggressive than NSCLC-AC [173]. Both NSCLC-AC and NSCLC-SCC are often diagnosed late, which contribute to the high death rates associated with them [170, 171].

Mutations in gene-encoding EGFR are the second most common oncogenic drivers in NSCLC-AC, behind mutations in the KRAS gene [174]. The two most common mutations in the NSCLC-associated EGFR gene are exon 19 deletions and the exon 21 point-mutation resulting in L858R which, combined, comprise ca. 90% of the EGFR-activating mutations found in NSCLC [174]. Historically, small molecule tyrosine kinase inhibitors (TKIs), such as, gefitinib, erlotinib, afatinib, dacomitinib, and the most advanced, osimertinib, have been used to treat EGFR-mutated NSCLC, but resistance against those inhibitors develops rapidly [174]. More recently, Rybrevant® (amivantamab), an EGFR × cMET-bispecific antibody, has been used successfully, in combination with Lazcluze™ (lazertinib, third-generation TKI), as first-line therapy to treat patients with NSCLC-AC with specific EGFR exon 19 deletions or exon 21 L858R substitution mutations [175]. The FDA approval for the subcutaneous version of Rybrevant® [61] will be described in more detail later in this paper.

### Datopotamab deruxtecan (Datroway®, aka Dato-DXd)

Dato-DXd, developed by Daiichi Sankyo and commercialized in collaboration with AstraZeneca, was fully approved by the FDA on 17 January 2025, for treatment of adult patients with unresectable or metastatic, HR-positive/HER2-negative (HR^+^/HER2^−^) BC, who have received only a single prior chemotherapy and endocrine-based therapy [26, 27]. Dato-DXd was also approved on 23 June 2025, for treatment of adult patients with locally advanced or metastatic EGFR-mutated NSCLC [26, 27]. The future for Dato-DXd as an anticancer drug looks bright, as its project peak sales are estimated to be in the $5 billion range (Table 1) [24]. This is important because it would mark yet another successful antibody, and more specifically ADC, for treating solid tumors, adding to the growing list of antibodies to finally find success in treating some of the more difficult-to-treat forms of cancer, e.g. solid tumors.

Dato-DXd is an anti-TROP-2, humanized IgG1κ ADC, in which the antibody is linked by a glycyl-glycyl-phenylalanyl-glycine (GGFG) tetrapeptide linker to the camptothecin derivative, deruxtecan, with an average DAR of 4 [25, 176]. Dato-DXd binds human TROP2 with an affinity of 0.74 nM and cross-reacts with TROP2 from nonhuman primates but not mice. Also, Dato-DXd does not bind to the highly similar protein, human TROP1, also known as EpCAM (epithelial cell adhesion molecule) [176].

The linker-warhead moiety is conjugated via thioether bonds to the antibody through four reduced cysteinyl residues (Fig. 3, Table 2). Dato-DXd is stable in circulation, but once it binds to TROP2 in the tumor microenvironment and is internalized, the GGFG linker is cleaved by lysosomal proteinases, particularly cathepsin B, to release the active cytotoxic moiety, an exatecan derivative (DXd) [176]. DXd inhibits Topoisomerase I, which ultimately results in DNA damage and immunogenic cell death, which is typically differentiated from classical apoptosis via migration of calreticulin to the cell surface and release of ATP and the alarmin protein, high-mobility group box 1 (HMGB1) [177]. Additionally, because DXd is highly membrane-permeable, it can easily diffuse out of the dying cell and enter adjacent tumor cells, killing them via a bystander effect [176]. The bystander effect is critical to its activity because solid tumors are notoriously heterogeneous and not all cells within the tumor will express the TROP2 target. By possessing the bystander killing MOA, Dato-DXd can also kill TROP2-negative cells in the tumor.

### Datroway® approval for hormone-positive, HER2-negative breast cancer

The efficacy of Dato-DXd for treatment of HR^+^/HER2^−^ breast cancer was evaluated in the TROPION-Breast01 (NCT05104866 [178]) trial, a multicenter, open-label, randomized trial of 732 patients with unresectable or metastatic HR^+^/HER2^−^ (IHC 0, IHC1^+^, or IHC2^+^/ISH^−^) breast cancer [179]. Based on results of the TROPION-Breast01 clinical trial [179], Dato-DXd was granted full approval for the treatment of advanced or metastatic HR^+^/HER2^−^ BC [180]. In that trial, progression-free survival (PFS) for patients in the treatment arm was 6.9 months as compared with 4.9 months in the chemotherapy arm (*P* < .0001) [179]. Additionally, the median duration of response (mDOR) was 6.7 months (95% confidence interval [CI], 5.6–9.8 months) vs 5.7 months (95% CI, 4.9–6.8 months) in the Dato-DXd vs chemotherapy arms, respectively [179]. There was no statistical difference in median overall survival (OS) for patients in the Dato-DXd cohort vs those receiving chemotherapy [179].

### Datroway® approval for previously treated NSCLC

Dato-DXd was granted accelerated approval for the treatment of patients with NSCLC who had previously been treated with a TKI or platinum, based on results from a pooled subgroup of patients from the single-arm, open-label TROPION-Lung05 phase 2 trial [181] and the TROPION-Lung01 phase 3 trial [182]. The accelerated approval was based on a pooled analysis of 114 patients, which had a 45% objective response rate (ORR), composed of a 40% partial response rate and a 4.4% complete response (CR) rate, as well as an mDOR of 6.5 months [183].

Dato-DXd is dosed Q3W via intravenous (IV) infusion at 6 mg/kg [26]. The average circulating half-life of the intact ADC is 4.8 d with a clearance of 0.6 L/d, while the half-life of the DXd active moiety is ~5.5 d, the combination of which allows for the Q3W dosing regimen. The circulating half-life of Dato-DXd and its released DXd is significantly greater than the circulating half-life of sacituzumab govitecan-hziy (Trodelvy®) and its free cytotoxin, SN-38, which are 16 and 18 h, respectively [26]. This increased stability allows for significantly less frequent dosing than Trodelvy®, which must be dosed weekly (QW) [95].

The pathway for DXd degradation is via CYP3A4, which is the most abundant cytochrome P450 in liver and most critical enzyme for drug metabolism [26, 184]. Nevertheless, CYP3A inhibitors such itraconazole did not appear to significantly alter the metabolism of DXd from trastuzumab deruxtecan in a previous study [184], so the expectation is that CYP3A inhibitors should not significantly change the catabolism of DXd from Dato-DXd [184].

## Emrelis® (telisotuzumab vedotin-tllv) – anti-CMET ADC

### cMET (mesenchymal–epithelial transition receptor)

The cMET receptor tyrosine kinase (RTK), also known as the Hepatocyte Growth Factor Receptor (HGFR), is a single-pass tyrosine kinase receptor essential for cell survival, growth, and wound repair. Initially produced as a ~170 kDa precursor protein, cMET is cleaved into a ~50 kDa α-subunit and a ~140 kDa β-subunit, which are connected by a disulfide bond [185]. This receptor is generally found on the surface of epithelial and endothelial cells, and when activated by its ligand, HGF, it initiates downstream signaling pathways such as PI3K, MAPK, and STAT3, which are involved in controlling cell growth, movement, invasion, and morphogenesis [185]. Mutations that activate cMET, often occurring in the tyrosine kinase domain, along with its overexpression or amplification, are key oncogenic drivers in some forms of NSCLC, gastric cancer, and papillary renal cell carcinoma [186]. The protein cMET is overexpressed in ~25%–70% of patients with nonsquamous (NSQ), EGFR-wild-type NSCLC, which frequently contributes to tumorigenesis and generally leads to a poor prognosis [186, 187]. This relatively common overexpression phenotype, identified via immunohistochemistry (IHC) typically using a 25%–50% tumor cell cutoff, is completely separate from cMET gene amplification or exon 14 skipping, which comprise ~2%–5% and 3% of NSCLC cases, respectively [186, 187]. One of the key outcomes of cMET overexpression is HGF-ligand independent receptor activation by oligomerization of the receptor [188].

Treatments for cMET-driven NSCLC depend largely on the type of cMET modification away from normal expression [186]. Several small molecule kinase inhibitors have been approved for treatment of cMET-modified tumors. One example is crizotinib (Xalkori®), a dual ALK and cMET phosphorylation inhibitor that works best in patients with high *MET* gene amplification and cMET copy numbers [186]. Others include capmatinib (Tabrecta®) and tepotinib (Tepmetko®), both of which block cMET phosphorylation to inhibit downstream signaling and are used for NSCLC tumors harboring *MET* exon 14 skipping [186]. As noted previously, amivantamab (Rybrevant®), a cMET × EGFR bispecific, high-ADCC (low-fucose) antibody, has been approved to treat NSCLC harboring EGFR exon 19 deletions, exon 20 insertions, or exon 21 mutation L858R [61], but it also appears to be effective in cMET exon-14 skipped cancer [189]. Very few options, however, have previously been available for those NSCLC patients with cMET protein overexpression [186–188].

### Emrelis® (telisotuzumab vedotin-tllv)

Emrelis® (telisotuzumab vedotin-tllv; Teliso-V), an ADC developed and sponsored by Abbvie, received accelerated approval from the FDA on 14 May 2025 for treatment of adults with locally advanced or metastatic nonsquamous NSCLC who exhibit high cMET receptor overexpression and have previously undergone systemic therapy [190]. Overexpression of cMET was defined as ≥50% 3^+^ staining as determined by the VENTANA MET (SP44) RxDx Immunohistochemistry (IHC) Assay developed by Roche that was approved concomitantly with Emrelis® [191].

Teliso-V is a first-in-class anti-human cMET humanized IgG1κ ADC conjugated through cysteinyl moieties to vedotin, composed of a cathepsin-B-cleavable maleimidocaproyl-valine-citrulline-*p*-aminobenzyl carbamate (mc-val-cit-PABC) linker and monomethyl auristatin E (MMAE), with an average DAR of 3 [192]. Upon binding the overexpressed cMET RTK, the ADC is internalized and the microtubule inhibitor cytotoxic payload, MMAE, is released in the lysosome via proteolytic cleavage of the linker [192]. This process leads to cell cycle arrest in the *G2/M* phase and induces apoptosis in cancer cells [192].

The FDA-accelerated approval of Teliso-V was supported by data from the Phase 2 LUMINOSITY clinical trial (NCT03539536) [193, 194], which studied, without a comparator arm, the efficacy and safety of Teliso-V in patients with varying degrees of advanced wild-type cMET overexpressing NSCLC [194]. Patients with the highest wild-type cMET protein overexpression (*n* = 78) who received Teliso-V demonstrated a 34.6% (95% CI: 24–46) ORR, an mDOR of 9.0 months (95% CI: 4.2–13), and a progression-free survival of 5.5 months (CI: 4.1–8.3 [194]. The most common adverse reactions (≥20%) were peripheral neuropathy, fatigue, decreased appetite, and peripheral edema [194]. As expected with an accelerated approval, Emrelis® is being further evaluated as a monotherapy head-to-head versus docetaxel in previously treated NSCLC patients with overexpressed wild-type cMET in an open-label randomized Phase 3 confirmatory global study (NCT04928846) [195].

As with many ADCs, the circulating half-life of intact drug, Teliso-V, is a relatively short, ca. 3 days, with an estimated clearance (CL) of 1.3 L/day [35]. Once released from the intact drug, the circulating half-life of the released MMAE moiety is ca. 4 days [35], with a clearance of 76 L/day [35]. These data support the Q2W IV dosing of Teliso-V [35].

The small-molecule, natural product warhead of Teliso-V, “vedotin,” is metabolized by CYP3A [35, 196], so there are potential concerns about drug–drug interactions (DDIs) with either CYP3A inducers or inhibitors and MMAE, or *vice versa*, i.e. the possibility of MMAE inhibiting the metabolism of other drugs metabolized by CYP3A4 [196]. Han *et al*. [196] demonstrated that the exposures of MMAE released from brentuximab vedotin were lowered in the presence of the strong CYP3A4 inducer, rifampin, and higher with the strong CYP3A4 inhibitor, ketoconazole. In that study, the safety profile of brentuximab vedotin did not appear to be affected by the presence of the perpetrating molecules. These results were corroborated for MMAE released from Teliso-V by Riad *et al*. [197], who used physiologically based PK modeling, followed by verification using clinical data, to predict that MMAE exposure was increased 43% in the presence of ketoconazole but was decreased 70% in the presence of rifampin. On the other hand, Han *et al*. [196] demonstrated that MMAE as perpetrator did not significantly alter the metabolism of the sensitive CYP3A substrate, midazolam.

Approximately 22% (60/269) of patients receiving Teliso-V developed antidrug antibodies (ADAs), and 23 of those (38% of total ADAs) were deemed to be neutralizing [35]. The presence of these ADAs reduced the clearance of, i.e. increased exposure to, Teliso-V by 17%, presumably through stabilization of the intact ADC in circulation [35]. This increased exposure not only resulted in improved efficacy but also came with greater safety risks [198].

## Exdensur® (depemokimab-ulaa)–long half-life anti-IL-5

### Interleukin-5

Interleukin 5 (IL-5) is a type-2 (T-helper-2; Th2) cytokine produced by T helper cells and mast cells [199]. Human IL-5 is a 4-helix bundle cytokine of 115 amino acids in length that is a member of the βc (common β chain) family of hematopoietic cytokines, along with IL-3 and GM-CSF [198]. IL-5 is a glycoprotein that forms active homodimers that bind to the heterodimeric IL-5 receptor complex, composed of IL-5Rα and βc, to drive eosinophil production (eosinopoiesis) in bone marrow, as well as promoting eosinophil maturation, activation, and survival in peripheral tissues [200]. One of the key natural biological functions of IL-5 is to facilitate eosinophil response to provide immunity to helminths [200].

IL-5, which is a central mediator in Th2 inflammatory conditions, has been linked to the development of various allergic responses, including allergic rhinitis and eosinophilic asthma, the latter in which there is a significant rise in eosinophils in the bloodstream, airway tissues, and induced sputum [199–201]. At the beginning of 2025, three mAbs targeting the IL-5 pathway had been FDA-approved to treat severe eosinophilic asthma (SEA), including the anti-IL-5 mAbs, Nucala® (mepolizumab, FDA-approved 2015 [202]) and Cinqair® (reslizumab, FDA-approved 2016 [203]), as well as the anti-IL-5Rα mAb, Fasenra® (benralizumab, FDA-approved 2017 [204]) [202–206]. In addition to the SEA indication above, mepolizumab is indicated for treatment of chronic obstructive pulmonary disease, eosinophilic granulomatosis with polyangiitis (EGPA), hypereosinophilic syndrome, and chronic rhinosinusitis with nasal polyps (CRSwNP) [202], while benralizumab is also indicated for EGPA [204].

### Eosinophilic asthma and chronic rhinosinusitis with nasal polyps

Eosinophils are granulocytes that make up 1%–6% of the total white blood cell count. Eosinophils play a significant role in asthma and other respiratory conditions, including a severe form of asthma known as “eosinophilic asthma.” When activated by Th2 cytokines (e.g. IL-5, IL-4, IL-13), eosinophils release toxic granules that can damage airway tissues, increase mucus, and cause bronchial hyperresponsiveness [199, 207]. High eosinophil counts (>400 cells/μl) in blood or sputum are associated with severe disease exacerbations. While eosinophilic asthma can exist without allergies, most patients with this subtype also have allergic asthma [207]. Additionally, type 2 inflammation associated with eosinophils is found in up to 85% of individuals with CRSwNP, where it is linked to more severe disease manifestations and symptoms [207].

Asthma impacts over 260 million individuals worldwide, with many still experiencing symptoms and flare-ups despite using high-dose inhaled corticosteroids, along with an additional controller or systemic corticosteroids [208]. In 2021, it was estimated that nearly 25 million people in the USA, or ca. 7.7% of the total population, had some form of asthma [209]. There are two fundamental forms of asthma, allergic asthma and “intrinsic” (nonallergic) asthma [210]. Allergic (atopic) asthma is typically an early onset or childhood form of asthma often accompanied by allergic rhinitis, often associated with elevated IgE levels, and typically associated with lower or variable eosinophil counts [210]. Allergic asthma typically responds to inhaled corticosteroids but not to anti-IL-5 antibodies [209, 210]. Intrinsic asthma, on the other hand, is a nonatopic disease that is not commonly triggered by typical allergens but is instead driven by underlying Th2 immune responses. Intrinsic asthma is typically associated with chronic airway inflammation driven by high eosinophil [207, 210] or, sometimes, neutrophil counts [211]. Additionally, CRSwNP and chronic rhinosinusitis without nasal polyps (CRSsNP) are often comorbidities with intrinsic asthma [207, 210]. A major subtype of intrinsic asthma is eosinophilic asthma, which is an adult-onset asthma (typically between ages 35 and 50) that is characterized by high (>150 cells/μl) to very high (>400 cells/μl) eosinophil counts [207, 212]. While only ca. 10% of all asthma cases are categorized as severe asthma, eosinophilic asthma comprises ~50%–60% of severe cases. Typically, eosinophilic asthma does not respond well to inhaled corticosteroids but can be treated with anti-IL-5 antibodies, indicating the link between the Th2 cytokine driver, the target cells, eosinophils, and the disease [201, 207, 212].

### Exdensur® (depemokimab-ulaa)

Exdensur® (depemokimab-ulaa), sponsored by GlaxoSmithKline, was approved by the FDA on 16 December 2025 as an add-on treatment of patients with SEA [60]. In the UK [213] and Europe [214], depemokimab has been approved for two indications: add-on maintenance for patients with SEA and add-on therapy for adults with severe CRSwNP that is not controlled with inhaled corticosteroids [213, 214]. Depemokimab (aka GSK3511294) is an anti-human IL-5, humanized IgG1κ antibody with its Fc CH2 region modified by M252Y, S254T, T256E, the now well-known “YTE” mutations [66] that significantly improve antibody half-life (Table 3) [206, 215].

Depemokimab was approved in the USA based on the results of the SWIFT-1/SWIFT-2 phase 3 clinical trials [216, 217], which focused on patients with severe asthma characterized by high eosinophil counts. SWIFT-1 (382 patients) and SWIFT-2 (380 patients) each were 52-week, placebo-controlled (randomized 2:1), double-blind, multi-center Phase 3 clinical trials designed to study the efficacy and safety of depemokimab as an add-on therapy (over standard of care) for control of SEA [216, 217]. In the SWIFT trials, the patients had a median eosinophil count of 310–340 eosinophils/μl and an average of 2.2–2.7 exacerbations during the previous year [216]. Patients treated with depemokimab experienced 58% and 48% reductions (SWIFT-1 and SWIFT-2, respectively) in the rate of asthma exacerbations over 52 weeks versus placebo [216]. Additionally, depemokimab-treated patients had fewer exacerbations requiring emergency room visits and/or hospitalization. Adverse reactions in the depemokimab-treated patients included relatively low incidences of upper respiratory tract infection, allergic rhinitis, influenza, arthralgia, and pharyngitis.

The UK (15 December 2025) and EU (17 February 2026) approvals for Exdensur® (depemokimab) also included data from the ANCHOR-1/ANCHOR-2 [217, 218] clinical trials, which focused on patients with severe asthma and CRSwNP [218]. ANCHOR-1 and ANCHOR-2 were 52-week, placebo-controlled (randomized 1:1), double-blind, multi-center Phase 3 clinical trials [217, 218]. ANCHOR-1 (143 depemokimab-treated versus 128 placebo subjects) and ANCHOR-2 (129 depemokimab-treated versus 128 placebo subjects) successfully met their co-primary endpoints, which included the change from baseline in the total endoscopic nasal polyp score at 52 weeks and the mean nasal obstruction verbal response scale (VRS) score from Weeks 49 to 52 [217, 218]. The combined treatment groups from both trials exhibited a −0.7 difference from placebo (95% CI −0.9 to −0.4; *P* < .001) for total endoscopic nasal polyps. For the mean nasal obstruction VRS score, the combined treatment groups from both trials demonstrated a −0.24 difference from placebo (95% CI −0.39 to −0.08; *P* = .003), indicating a significant reduction in disease burden for patients with CRSwNP [217, 218]. Additionally, depemokimab was found to have a safety profile in treated patients very similar to that of subjects in the placebo arm.

Depemokimab is administered by a healthcare professional twice annually at 100 mg (in 1 ml) SC. The time from dose to maximal systemic concentration (*T*_max_) averaged 14 days, which is significantly longer than found with most SC-dosed antibodies [59]. The estimated terminal circulating half-life after SC dosing was an average of 48 (±4.7 SD) days, and the estimated clearance rate was 0.092 L/day [59], which supported the Q6M dosing regimen. This half-life is somewhat shorter than either the mean (63 days) or median (71 days) for the group of half-life extended antibodies analyzed in Table 3, whereas the clearance was between the median and mean values for the subset of antibodies for which those data were available (Table 3).

The incidence of ADAs against depemokimab in treated patients was 10% (66/691), and of those, 6% (4/66) developed neutralizing antibodies. Nevertheless, the ADAs did not result in clinically significant effects on the PK, PD, safety, or efficacy of the drug [59].

## Enflonsia™ (clesrovimab-cfor)–anti-RSV

### Respiratory syncytial virus

Respiratory syncytial virus (RSV) is a negative-sense, single-stranded virus that frequently causes seasonal respiratory infections, particularly affecting the lower respiratory tract [219]. It poses a significant threat to young children, especially infants, as well as to elderly people with comorbidities. RSV is the leading cause of respiratory illnesses in young children, with a global incidence rate of 9.5% and a mortality rate of 2.2% [219, 220]. The mortality rate is even higher among infants who are immunocompromised or have additional risk factors. Unlike many other viruses, RSV can reinfect individuals within the same season, making it particularly challenging to manage. Initial efforts to protect infants from RSV involved administering RSV-specific IVIG to children under 24 months who were born prematurely or had respiratory issues or other high-risk factors [221]. However, this method was eventually found to be ineffective [221], leading to the development of a highly potent, specific mAb for RSV prophylaxis. Synagis® (pavilizumab), a humanized antibody targeting the A-antigenic site of the RSV-F protein, was approved by the US FDA in 1998 for prophylaxis against RSV infection [222]. Due to its limited half-life and PD effects, pavilizumab is administered intramuscularly Q4W during the RSV season to prevent severe lower respiratory tract disease in high-risk infants [222]. For two decades, both AstraZeneca, the sponsor of Synagis®, and Merck have been trying to develop a more effective and longer-lasting version of Synagis® [223], but success has been elusive until recently, when AstraZeneca received approval in 2023 for Beyfortis® (nirsevimab) for long-term prophylaxis against RSV infection [11, 224, 225].

### Clesrovimab

The US FDA granted full approval on 6 June 2025, for Enflonsia™ (clesrovimab-cfor), an anti-RSV mAb developed by Merck, for prevention of RSV lower respiratory tract disease in newborns and infants born during or entering their initial RSV season [39]. Clesrovimab (previously known as MK-1654 and RB-1 [226]) is a fully human IgG1κ mAb targeting RSV F protein, isolated from human memory cells [226]. Clesrovimab neutralizes RSV, providing passive immunity, preventing the fusion of viral and cellular membranes and subsequent viral entry. Clesrovimab contains the well-known YTE triple amino acid substitution (M252Y/S254T/T256E) in its Fc region, which enhances its binding to FcRn, thereby significantly prolonging its serum half-life [40, 226, 227].

Clesrovimab binds to a conserved epitope on antigenic site IV of RSV F protein and, while RB1 was isolated using post-F protein as the antigen, clesrovimab interacts with both the RSV-A prefusion and postfusion F glycoproteins, with affinities (*K*_D_) of 71 and 480 pM, respectively, functioning as a fusion inhibitor [226]. Clesrovimab also binds the F protein of RSV B viruses and neutralizes RSV B. In cell-based neutralization assays, clesrovimab successfully neutralized 47 RSV clinical isolates isolated from various North American locations between 1987 and 2016 [226]. The median neutralization IC_50_ values for clesrovimab-treated RSV A and RSV B isolates were 3.71 and 4.46 ng/ml, respectively, [226] demonstrating broad neutralizing activity. These values are similar to the neutralization values previously obtained with nirsevimab, which neutralized panels of RSV A and RSV B isolates with median IC_50_ values of 3.1 and 3.0 ng/mL, respectively [228]. It has been shown that Fc function is not required for clesrovimab neutralization activity [226], similar to what was found with nirsevimab [128, 229].

Clesrovimab was approved by the FDA based on two large clinical trials, a phase 2b/3 double-blind, randomized, placebo-controlled trial, CLEVER (MK-1654-004) [230] (NCT04767373 [231]) and a phase 3, randomized, partially blinded, palivizumab-controlled trial, SMART (MK-1654-007) [232] (NCT04938830 [233]). The CLEVER trial studied near-full-term to full-term (gestational age ≥ 29 weeks) infants from their birth up to 1 year entering their first RSV season. The infants were randomized 2:1 against placebo (clesrovimab, *N* = 2411; placebo, *N* = 1203) and the primary endpoint, measured over 5 months after dosing, was incidence of RSV-associated cough, difficulty breathing, wheezing, and other respiratory distress symptoms [known as Medically Attended Lower Respiratory Infection (MALRI)]. The incidence rate of MALRI over the 5-month study period was 0.026 (60/2411) versus 0.065 (74/1203) for the clesrovimab-treated infants and placebo control infants, respectively, yielding 60.5% efficacy [38, 230]. Over the same period, 9/2411 in the clesrovimab arm were hospitalized (0.004 incidence rate) vs 28/1203 (0.065 incidence rate) hospitalized in the placebo arm, resulting in 84.3% efficacy [38, 230].

The SMART trial studied preterm infants born at gestational age ≤35 weeks, or infants with chronic lung disease or hemodynamically significant congenital heart disease from their birth up to 1 year entering their first RSV season. In the SMART trial, the moderate preterm neonates were treated with either clesrovimab (*N* = 446), as a single 105 mg (150 mg/ml × 0.7 ml) dose over the 5-month study period, or 15 mg/kg pavulizumab (*N* = 450) dosed monthly for up to 5 months [38, 232]. The primary endpoint was RSV-associated MALRI. In this study, both the incidence rates of MALRI and hospitalizations over the 5-month study period were highly similar between the clesrovimab (MALRI, 3.6%; hospitalization, 1.3%) versus pavulizumab (MALRI, 2.9%; hospitalization, 1.5%) arms [38, 232].

A significant concern for using mAbs as prophylactic treatments for viral infections is the potential for antibody-resistant mutants to rise, making the prophylaxis potentially ineffective [234]. In a recent detailed study, the overall incidence of mutations leading to resistance for either nirsevimab or clesrovimab was found to be quite low [234]. Nevertheless, RSV mutants resistant to clesrovimab have been found both in laboratory studies and in clinical trials [226, 230, 232, 234]. Laboratory-derived mutants of RSV A and RSV B resistant to clesrovimab were obtained via repeated passage through cell culture in the presence of clesrovimab. Four variants emerged after six passages with RSV A, and one variant appeared after nine passages with RSV B [226]. The variants carried specific substitutions at the clesrovimab binding site, including G446E, S443P + K445N, S443P + G446E, or S443P for the four RSV A variants, and an S443P substitution for the RSV B variant [226]. These variants exhibited reductions in susceptibility to clesrovimab of over 3800-fold for RSV A and over 360-fold for RSV B [226]. In clinical trials, variants were observed at a greater frequency in the clesrovimab arm (ca. 9.6%) than in the placebo arm (1.3%) [38, 230]. Most of the variants had substitutions at G446: for RSV A, the top variants were G446E,R,W, resulting in loss of susceptibility of >2941-fold, and for RSV B, the most significant variants were G446E,R, resulting loss of susceptibility >1299-fold [38, 226]. Those variants that lost susceptibility to clesrovimab were still sensitive to the other anti-RSV mAbs, pavulizumab and nirsevimab, as well as *vice versa* [38].

Clesrovimab is administered via a single intramuscular (IM) injection of 105 mg, which was shown to provide protection for up to 5 months. The median time to reach maximum concentration (*T*_max_) was 6.5 d, and estimated apparent clearance was 0.0197 L/day for a typical infant weighing 5 kg, resulting in a terminal half-life of ca. 44.0 days [38]. Like other biological therapies, patients may develop antibodies against clesrovimab over time. ADAs were detected in 5%–6% of participants after 150 days and 12%–13% after 240 days [38, 235].

## Lynozyfic® (linvoseltamab-gcpt)–BCMA × CD3-bispecific TCE for myeloma

### B-cell maturation antigen

B-cell maturation antigen (BCMA), also known as CD269 and tumor necrosis factor receptor superfamily 17 (TNFRS17), is a single-pass transmembrane glycoprotein receptor primarily found on plasmablasts, long-lived plasma cells, and multiple myeloma (MM) cells but not on naïve B cells or germinal-center B cells [236]. BCMA is typically found on the cell surface as a monomer, but it trimerizes into its active form upon ligand binding [237]. The exodomain can be shed by cleavage with γ-secretase, resulting in circulating soluble BCMA (sBCMA), which is a hallmark of active MM [238].

BCMA has 184 amino acid residues and an approximate molecular mass of 20 200 Da, with a single N-terminal exodomain of ~50–54 amino acid residues [236]. The exodomain functions as a receptor for two soluble ligands, APRIL and B-cell-activating factor (BAFF; also referred to as B-lymphocyte stimulator [BLyS], CD257, TNFSF138).

BCMA is one of three receptors, along with BAFF-R and TACI (transmembrane activator and calcium-modulating cyclophilin ligand interactor), for binding the ligand BAFF [239]. Another ligand for BCMA, called APRIL, has also been identified that binds BCMA and TACI but not BAFF-R and is also notably elevated in MM [239]. APRIL binding to BCMA occurs with a high affinity of ca. 11 nM, whereas BAFF binding occurs in the range of 8 μM, ca. 1000-fold lower affinity than APRIL [240]. Note that the newly approved anti-APRIL antibody, Voyxact® (sibeprenlimab), will be discussed in the next section of this review.

BCMA is a classic low-copy-number target; in a classic study across multiple subjects and patients, BCMA had a median of 673 (range, 189–1713) and 1479 (range, 42–14 055) antibody-binding capacity (ABC) units (approximate copy number/cell) for normal plasma cells and MM cells, respectively [241]. This is likely why no ADCC-driven IgGs have been successfully developed to treat MM, as the ADCC mechanism of action (MOA) typically requires antigen densities of at least 10 000 and typically over 20 000 receptors per cell for good NK-mediated killing, depending on other factors such as the IgG fucose level, FcγR polymorphisms, NK-cell activation levels, epitope on target, and IgG affinity [242].

BCMA, as its name suggests, supports B-cell proliferation, development, and long-term survival, depending on stage of maturation and binding by its ligands APRIL or BAFF [239]. In recent years, BCMA has been a heavily studied target for MM [236] and more recently for possible use in certain autoimmune diseases such as generalized myasthenia gravis (gMG), especially using newer, more potent technologies such as bispecific antibody TCEs, autologous chimeric antigen receptor (CAR)–T cells, and now, *in vivo* CAR-T cells [243].

### Multiple myeloma

MM, historically referred to as Kahler’s disease, represents ~1.8% of all human cancers, with around 32 000 new cases reported in the USA in 2020 [244]. This condition, which is becoming more common, is a B-cell cancer resulting from the unchecked growth of plasma cells within the bone marrow. MM cells generate M proteins, which are mAbs or their fragments that lack a specific function [244]. The initial phase of plasma cell transformation into MM is termed monoclonal gammopathy of undetermined significance (MGUS), which can progress to smoldering (or asymptomatic) “SMM,” eventually leading to MM. In MM, the proliferating MM cells displace normal red and white blood cells in the bone marrow, causing anemia and infections, respectively [245]. The buildup of MM cells and their products in the bone marrow also results in bone pain, fractures (particularly in the spine), bone loss, and potential organ failure [246].

Since its approval in 2015, the anti-CD38 antibody Darzalex® (daratumumab) has been the standard antibody treatment for MM [247]. Although daratumumab has been a valuable addition to the treatment options for MM, many patients either do not respond to the drug or develop resistance to it. Furthermore, daratumumab is known to cause the destruction of healthy CD38-positive NK and T cells (known as “fratricide”), which could theoretically diminish its efficacy [248]. Consequently, there has been a considerable effort to identify alternative antibody targets, such as BCMA, CD33, and GPRC5D, and antibody-like formats such as TCEs and CAR-Ts for treatment of MM. These new antibody-based MM treatments include: Blenrep® (belantamab mafodotin), an ADC that consists of an afucosylated anti-BCMA IgG linked via a noncleavable linker to monomethyl-auristatin F (J6M0-mcMMAF), approved on 5 August 2020 [249] (Table 2); Tecvayli® (teclistamab-cqyv), a BCMA × CD3ε-bispecific TCE approved on 25 October 2022 [250]; Elrexfio® (elranatamab-bcmm), a second BCMA × CD3ε TCE approved on 14 August 2023 [251]; Abecma® (idecabtagene vicleucel), an anti-BCMA autologous CAR-T, approved on 6 March 2021, and Carvykti® (ciltacabtagene autoleucel), a biparatopic anti-BCMA autologous CAR-T, approved on 28 February 2022. Now, Lynozytic® (linvoseltamab-gcpt) has become the third FDA-approved TCE and the fifth antibody-based biologic targeting BCMA for treatment of MM [252]. Additionally, another bispecific TCE, Talvey® (talquetamab), targeting the myeloma cell–enriched target, G protein–coupled receptor, class C, group 5, member D (GPRC5D), and CD3ε to treat MM [253], was approved on 9 August 2023.

In the first paper of this series, a detailed table was provided comparing the efficacy and safety of the four MM-targeting TCEs, including the three FDA-approved TCEs and the then-Phase 3 stage, linvoseltamab, along with the market-leading MM antibody, daratumumab (Darzelex®), and other antibodies [11]. As of 2025, the total worldwide market for MM-treating antibody-based biologics, including the two approved BCMA CAR-Ts, has increased to $18.7 billion in 2025 (Fig. 2).

### Lynozyfic® (linvoseltamab-gcpt) a new BCMA × CD3ε-bispecific TCE

Lynozyfic® (linvoseltamab-gcpt), sponsored by Regeneron, was granted accelerated approval by the FDA on 2 July 2025 for the treatment of adult patients with relapsed or refractory (R/R) MM who have received at least four prior lines of therapy, including a proteasome inhibitor (e.g. Velcade® [bortezomib]), an immunomodulatory agent (e.g. an IMiD such as lenalidomide), and an anti-CD38 mAb [45, 252]. Linvoseltamab (aka REGN5458) is a fully human, Fc-silenced IgG4κ-based bivalent, bispecific BCMA × CD3ε TCE with a stabilized hinge (S228P) [67, 68] that was derived from two “parental” mAbs, an anti-BCMA mAb and an anti-CD3ε mAb, and combined to form a bivalent, bispecific heterodimeric IgG [254]. Both sides of the heterodimeric Fc were altered to reduce the antibody’s ability to bind Fc receptors and complement (Table 1) [254]. Additionally, modifications (H435R, Y436F, L445P) were made to one of the heavy-chain Fc regions to remove protein A binding to improve the purification process [254, 255].

Linvoseltamab received FDA approval based on findings from the open-label LINKER-MM1 study [256] (NCT03761108 [257]), a Phase 2 study in which 117 patients with R/R MM were administered 200 mg of linvoseltamab QW for 14 weeks and then biweekly (Q2W) thereafter. Some patients who attained a very good partial response or better by the 24th week were switched to a Q4W regimen. With a median follow-up period of 14.3 months, the ORR was 71%, with 52% of the patients achieving a CR [256]. The mDOR was 29.4 months, and within the time period reported, the estimated median PFS had not yet been reached [256]. These ORR, CR, and mDOR data were improvements over similar data obtained with the other BCMA × CD3ε TCE antibodies, teclistamab and elranatamab, which exhibited ORRs of 62 and 58% and CRs of 28 and 29%, respectively, and an mDOR for teclistamab of 9 months [11].

All of the BCMA × CD3ε TCEs have exhibited significant AEs such as cytokine release syndrome (CRS) and neurological toxicity, including immune effector cell–associated neurotoxicity (ICANS). CRS (all grades combined) was observed in 46% of patients treated with linvoseltamab, most of which were grade 1 or 2 [256]. Since IL-6 is a major driver of AEs associated with CRS, it has been recommended that tocilizumab be provided prophylactically to patients treated with linvoseltamab and the other BCMA × CD3ε TCEs [258]. Additionally, ICANS was reported in 7.7% of patients receiving 200 mg doses of linvoseltamab. Both of these AE rates were in the same ranges as obtained with the other BCMA TCEs [11]. About 75% of patients treated with linvoseltamab experienced infections [256]. Linvoseltamab received black box warnings in its label for CRS and ICANS.

Linvoseltamab is dosed 200 mg IV QW, Q2W, or Q4W as noted above. While the circulating half-life of linvoseltamab has not been made readily available, its clearance rate was shown to be 0.43 L/d [45], which is ~4–10-fold higher than clearance rates of half-life extended antibodies (Table 3). The circulating half-life was long enough to support the Q1W, Q2W (after 14 weeks), and, for some patients, Q4W dosing. Only 1% of patients receiving linoseltamab developed ADAs, and those did not appear to affect efficacy or PK of the drug [45].

## Voyxact® (sibeprenlimab-szsi) for IgA nephropathy

### APRIL (A PRoliferation-Inducing Ligand)

APRIL (aka CD256, TNFSF13, TALL2, TRDL1) is an unusual 26 kDa transmembrane protein that is cleaved intracellularly in the Golgi apparatus by furin and released extracellularly as a soluble ligand, where it trimerizes into its active form [259]. APRIL also can form heterotrimers with BAFF [239]. As noted in the previous section, trimerized APRIL has two receptors, TACI (TNFRSF13b), to which it binds with 11 nM affinity to regulate B-cell maturation, survival, and antibody production, and BCMA (TNFRSF17), to which it binds with an affinity of 1.3 nM to promote survival of plasma cells in bone marrow [239, 240, 260]. APRIL is expressed by T cells, B cells, dendritic cells, monocytes, and macrophages and, in addition to its critical role in plasma cell survival, it promotes IgA class switching, and downregulation of T-cell-dependent antibody responses [261]. Additionally, APRIL can bind BCMA on myeloma cells to trigger signaling pathways (e.g. NF-κB, AKT, and MAPK) that promote tumor growth, survival, and drug resistance [262].

### IgA nephropathy

IgA nephropathy (IgAN), also known as synpharyngitic glomerulonephritis or Berger disease, is a disease of the kidney and the immune system in which the glomeruli of the kidney become inflamed. IgAN was first diagnosed as a unique disease in 1968 and now is recognized to comprise as many as 2.5 cases/100 000 individuals annually worldwide (e.g. ca. 200 000 cases) [263]. The highest rate of IgAN by country was found in Australia, where the incidence was reported to be 10.5 cases/100 000, whereas the lowest rate was found in South Africa at 0.06 cases/100 000 persons [264]. The inflammation is caused by accumulation of galactose-deficient IgA1 (Gd-IgA1), an abnormal, “pathogenic” form of IgA [265].

The current concepts for development of IgAN are described by the 4-hit model [261, 266]. Hit-1 is the formation of Gd-IgA1 by plasma cells [261]. APRIL drives Hit-1 via its interaction with both TACI and BCMA to promote class-switching of B cells to produce Gd-IgA1, as well as enhancing the proliferation of the Gd-IgA1-producing plasma cells [261, 267, 268]. Hit-2 is characterized by recognition of the overproduced Gd-IgA1 by the immune system as foreign, resulting in the production of pathogenic anti-Gd-IgA1 autoantibodies [261, 266]. Hit-3 results by the autoantibodies and Gd-IgA1 forming immune complexes that circulate in the bloodstream and deposit in kidney glomeruli [261, 266]. Hit-4 results from the deposition of the immune complexes in the glomerular mesangium where they activate the complement system to drive inflammation, mesangial cell activation, inflammatory cytokine release, and ultimately, end-stage kidney disease [261, 265, 267]. Current evidence suggests that APRIL-induced Gd-IgA1-producing plasma cells originate in mucosa-associated lymphoid tissues such as the tonsils (NALT) and the gut (GALT) wherein chronic stimulation by microbes induced APRIL triggers the differentiation of B cells into Gd-IgA1-secreting plasma cells [261].

### Voyxact™ (sibeprenlimab-szsi) an anti-APRIL antibody

Voyxact™ (sibeprenlimab-szsi), a first-in-class humanized anti-APRIL IgG2κ antibody, was granted accelerated approval by the FDA on 25 November 2025, for the reduction of proteinuria in adults with primary IgAN who are at risk for disease progression [52, 53]. Sibeprenlimab, formerly known as VIS649, was discovered and developed by Visterra, Inc., a wholly owned subsidiary of Otsuka. By neutralizing APRIL, sibeprenlimab reduces the levels of circulating IgA and Gd-IgA1, inhibiting the development of IgAN at the Hit-1 stage. The reduction of Gd-IgA1 lowers the potential for formation of immune complexes and associated inflammation, ultimately resulting in better kidney function and lowered proteinuria [265–267].

Sibeprenlimab was granted accelerated approval based on the results from a preset interim analysis of the Phase 3 Visionary clinical trial [269, 270] (NCT05248646 [271]), a randomized, double-blind, placebo-controlled, multicenter, global study of 510 patients with IgAN to evaluate the efficacy and safety of sibeprenlimab [269–271]. The patients were randomized 1:1 with half receiving 400 mg of sibeprenlimab administered SC Q4W compared to the other half who received placebo. The primary endpoint was the relative change from baseline in the urinary protein-to-creatinine ratio (uPCR; i.e. “proteinuria”) after 9 months of treatment [269–271]. Proteinuria is a widely accepted surrogate endpoint for kidney function in trials of IgAN [272]. The secondary endpoint was the comparison in treated versus placebo of the estimated glomerular filtration rate (eGFR) after ~24 months of treatment [269–271].

In an interim analysis of 320 patients after 9 months of study, a significant reduction in 24-h uPCR with sibeprenlimab-treated patients was demonstrated, with minus-50.2% as compared with plus-2.1% for those who received placebo, which corresponded to a calculated difference in uPCR of 51.2% (96.5% CI, 42.9%–58.2%; *P* < .001) [52, 270]. After 11 months of study, the levels of APRIL and pathogenic Gd-IgA1 in the sibeprenlimab-treated group were reduced from baseline by 95.8% and 67.1%, respectively [52, 270].

Sibeprenlimab is delivered Q4W SC using a single-dose, prefilled syringe, either self-administered or administered by a caregiver. The *T*_max_ after SC dosing is 8 d, with a bioavailability of 92% [52]. The mean terminal half-life of sibeprenlimab was 9.3 d with a clearance rate of 0.206 L/day [52]. At 4 weeks, a dose of 6 mg/kg (roughly equivalent to the 400 mg dose for a 70 kg subject) resulted in serum levels of sibeprenlimab of ~60 μg/ml (calculated from data in Mathur *et al*. [273]), which is 10%–20% above the *in vitro* IC_50_ values for blocking APRIL binding to both TACI and BCMA [274]. Even with this tight ratio, single 400 mg doses given SC to subjects in early clinical trials resulted in complete suppression of APRIL for up to 6 weeks [275], supporting the Q4W dosing regimen.

The overall safety profile for patients in the Phase 3 Visionary clinical trial treated with sibeprenlimab was found to be similar to that for patients given placebo [52, 270]. About 34% (88/256) of patients receiving sibeprenlimab developed ADAs, 24% (21/88) of which were neutralizing [52]. The ADAs significantly reduced exposure to sibeprenlimab and lowered the uPCR outcomes, but the significance of those differences was stated to be unknown at the time of publication [52].

## Andembry® (garadacimab-gxii) - anti-factor XIIa for HAE

### Factor XIIa

The coagulation cascade (aka secondary hemostasis) is a series of biochemical steps that promotes blood stability, preventing both excessive bleeding and unnecessary clot formation under normal circumstances. This cascade involves the sequential activation of clotting factor enzymes, each of which circulates in its inactive “zymogen” form until activated, that control the clotting process [276]. The coagulation cascade begins with two separate initiation pathways, the intrinsic and extrinsic pathways, which merge at the Factor Xa (FXa) to enter the third, or common, pathway [276].

**Figure 4:**
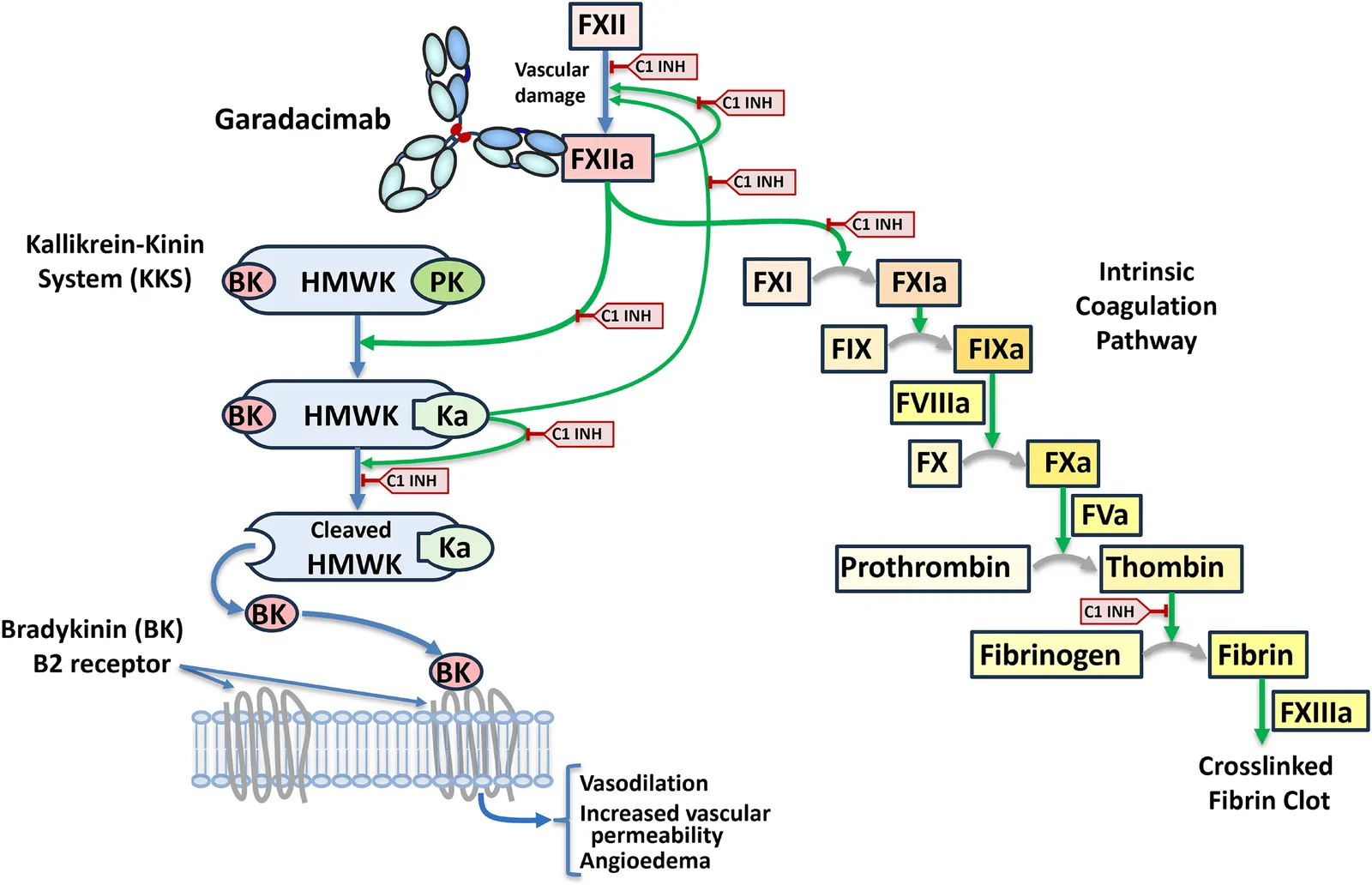
The dual role of Factor XIIa (FXIIa) in activating both the intrinsic coagulation pathway and the kallikrein–kinin pathway. The zymogen FXII is activated to FXIIa as a result of vascular damage. FXIIa then activates two pathways, the kallikrein–kinin system (KKS, left side) and the intrinsic coagulation pathway (right side). Garadacimab binds and neutralizes the enzymatically active FXIIa to inhibit activation of both pathways. Left side: Similarly, FXIIa can activate high-molecular-weight kininogen (HMWK)-bound prekallikrein (PK) to kallikrein (Ka), which catalyzes the conversion of the HMWK to cleaved HMWK, releasing the potent pro-inflammatory, nociceptive vasoactive nonapeptide, bradykinin (BK). This is the core set of reactions in the KKS [278, 279]. BK then binds to its GPCR B2, causing vasodilation, increased vascular permeability, and angioedema, hallmark components of HAE. C1-INH inhibits this pathway at multiple points as shown in the figure, as noted with boxed arrows. Right side: FXIIa activates Factor XI (FXI) to FXIa, which then continues the activation factors in the coagulation cascade until fibrinogen is converted to the insoluble fibrin mesh to promote clot formation [276, 277]. As noted with boxed arrows, C1-INH also inhibits this process at multiple sites, including FXII activation, FXIIa activation of FXI, and the terminal reactions in the common coagulation pathway. The green arrows indicate feedback activation steps.

When the vascular endothelium is damaged, exposing the negatively charged subendothelial collagen or activating platelets to reveal negatively charged phosphatidylserine, the intrinsic coagulation pathway is triggered through the activation of the zymogen Factor XII (FXII; also known as Hageman Factor) to FXIIa (Fig. 4) [276, 277]. FXIIa initiates two distinct physiological processes in the same biochemical network, the intrinsic coagulation pathway and the kallikrein–kinin system (KKS) to promote clotting and inflammation, respectively [278, 279] (Fig. 4).

FXIIa initiates the intrinsic coagulation pathway by activating Factor XI to Factor XIa, which then activates Factor IX to Factor IXa (FIXa), which, together with Factor VIIIa, activate Factor X (FX) to Factor Xa on the cell membrane surface [277]. FXa then combines with Factor Va (FVa) to form prothrombinase. Prothrombinase converts prothrombin into thrombin, which subsequently transforms fibrinogen into insoluble fibrin, which cross-links and stabilizes platelet aggregates at the site of vascular injury, forming a clot [276, 277] (Fig. 4).

### Hereditary angioedema

FXIIa activates the KKS by transforming plasma prekallikrein (PK), which is noncovalently bound to the nonenzymatic high-molecular-weight kininogen (HMWK), into active kallikrein [279] (Fig. 4). Kallikrein then cleaves HMWK to release the pro-inflammatory peptide, bradykinin, which binds to its G-coupled protein receptors (GPCRs), constitutively expressed B2 and inflammation-induced B1, on small blood vessels to induce swift vasodilation and enhance vascular permeability, leading to localized tissue swelling known as angioedema [279]. This localized, but potential life-threatening swelling that often impacts the face, lips, tongue, and airways is known as HAE [280].

C1 esterase inhibitor (C1-INH) is a suicide inhibitor that serves as the primary natural regulator of the KKS, also known as the contact system (Fig. 4). A member of the serpin (serine protease inhibitor) family, C1-INH prevents the excessive production of bradykinin from HMWK [281]. Additionally, C1-INH is a critical regulatory component of the classical complement pathway by blocking the activities of C1r and C1s [282, 283]. C1-INH also regulates the lectin pathway for complement activation by inactivating MASP-1 and MASP-2 [282, 283], and it also appears to regulate the alternative complement pathway by binding to C3b and preventing its interaction with factor B [284]. Figure 4 shows several points of contact where C1-INH functions to prevent HAE [283].

HAE is a rare genetic condition that affects roughly 2 individuals per 100 000 people [280]. HAE is caused by deficiency of C1-INH, and attacks can last up to 2–5 days from onset. Since HAE is a disease caused by the absence of C1-INH, the first treatments for HAE were C1-INH replacement proteins [285]. C1-INH replacement proteins, purified from human plasma, include Cinryze®, FDA-approved in 2008 as an IV-administered prophylactic to prevent HAE attacks [285], Berinert®, approved as an IV-administered protein in 2009 for treatment of HAE attacks [285], and Haegarda®, approved in 2017 as an SC-administered prophylactic to prevent HAE attacks [285]. A recombinant form of human C1-INH, Ruconest®, produced in transgenic rabbits, was approved in 2014 for prevention of HAE attacks [285]. These proteins, however, have a relatively short half-life of 30–60 h and limited usefulness in the treatment of HAE [286], so efforts have been made over the years to find novel approaches to treat HAE [287]. Those efforts have finally borne fruit, as significant advancements in the treatment of HAE have been made recently. In 2025, the FDA approved three new treatments for HAE within a short span, significantly improving both preventive and immediate care for the disease. The first was Andembry® (garadacimab), approved in June 2025 as the first mAb that targets activated Factor XIIa for the prevention of HAE attacks [42, 43]. Andembry® is administered via a QM (monthly) SC injection [42, 43]. Ekterly® (sebetralstat), approved in July 2025, is a rapid-acting oral plasma kallikrein inhibitor designed for the immediate, on-demand treatment of acute HAE attacks, enabling prompt intervention [288]. Finally, Dawnzera® (donidalorsen), which was approved in August 2025, is an RNA-targeted antisense oligonucleotide that reduces prekallikrein production by the liver, providing long-term prevention through SC injections Q4W or Q8W [289].

### Andembry (garadacimab-gxii)

Andembry® (garadacimab-gxii), sponsored by CSL Behring, was approved on 16 June 2025, as a prophylactic to prevent attacks of HAE. The label, however, specifically stated that garadacimab is not indicated for treatment of sudden, acute HAE attacks. Garadacimab (aka CSL312) is a first-in-class human anti-FXIIa IgG4λ with a stabilized hinge (S228P) [67, 68] that binds the catalytic domain of FXIIa and neutralizes its enzymatic function [290]. Since FXIIa is the initial factor activated in the contact system, leading to bradykinin production, neutralization of FXIIa activity prevents the formation of bradykinin, helping to reduce the inflammation and swelling associated with HAE attacks. One notable benefit of garadacimab is its ability to inhibit FXIIa, thereby blocking the pathway right from the beginning, in contrast to other HAE treatments that target mediators further down the line.

Garadacimab was approved based on results from the VANGUARD trial [291] (NCT04656418 [292]), a multicenter, randomized, double-blind, parallel-group study, which evaluated the efficacy and safety of garadacimab as a preventive treatment for individuals with HAE [291]. Patients were randomized in a 3:2 ratio to receive SC doses QM of garadacimab (*n* = 39) or placebo (*n* = 25). Garadacimab treatment was initiated with a 400 mg loading dose followed by QM 200 mg SC garadacimab. After 6 months of treatment, the patients in the garadacimab arm averaged 0.27 (95% CI 0.05–0.49) HAE exacerbations per month, compared to the placebo group, which had a mean of 2.01 (95% CI 1.44–2.57; *P* < .0001) [291]. The percentage difference was calculated to be a minus-87% (95% CI −96 to −58; *P* < .0001). Moreover, the median number of monthly HAE attacks for the garadacimab arm was 0 (IQR 0.00–0.31) versus 1.35 (IQR 1.00–3.20) for patients given placebo [291].

Garadacimab was dosed SC with a *T*_max_ of 6 days, a circulating half-life of 17 days, and an apparent clearance of 0.48 L/d [42], which supported QM dosing. Garadacimab was well tolerated with few significant safety signals. The most frequently observed garadacimab-related AEs included upper respiratory tract infections, nasopharyngitis, and headaches [42]. Inhibition of FXIIa by garadacimab did not result in an increase in bleeding or thromboembolic events [291]. In the Vanguard trial [291], only 1 of the 39 patients treated with garadacimab showed evidence of ADAs, and in that single response, no effects were observed on PK, PD, or safety of garadacimab [42].

## Yartemlea (narsoplimab-wuug) anti-MASP-2 for TA-TMA

### MASP-2 (mannan-binding lectin-associated serine protease-2)

The complement system has three initiation pathways: (i) the classical pathway initiated by binding of C1q to antibodies opsonizing a target cell or virus; (ii) the alternate pathway, initiated by C3/Factor B interaction in the absence of antibodies; and (iii) the lectin pathway, initiated by the binding of Pattern Recognition Molecules (PRMs) to sugars or other patterned residues on the surfaces of microorganisms such as bacteria, viruses, and fungi [293]. The lectin pathway functions independently of antibodies, serving as an initial innate defense mechanism against pathogens [294]. There are at least nine families of Lectin pathway PRMs include Mannose-Binding Lectin (MBL), which binds mannose, glucose, and other sugars, Ficolins, which target acetylated residues rather than just sugars, and Collectins, which are soluble proteins that also recognize microbial oligosaccharides [294].

**Figure 5:**
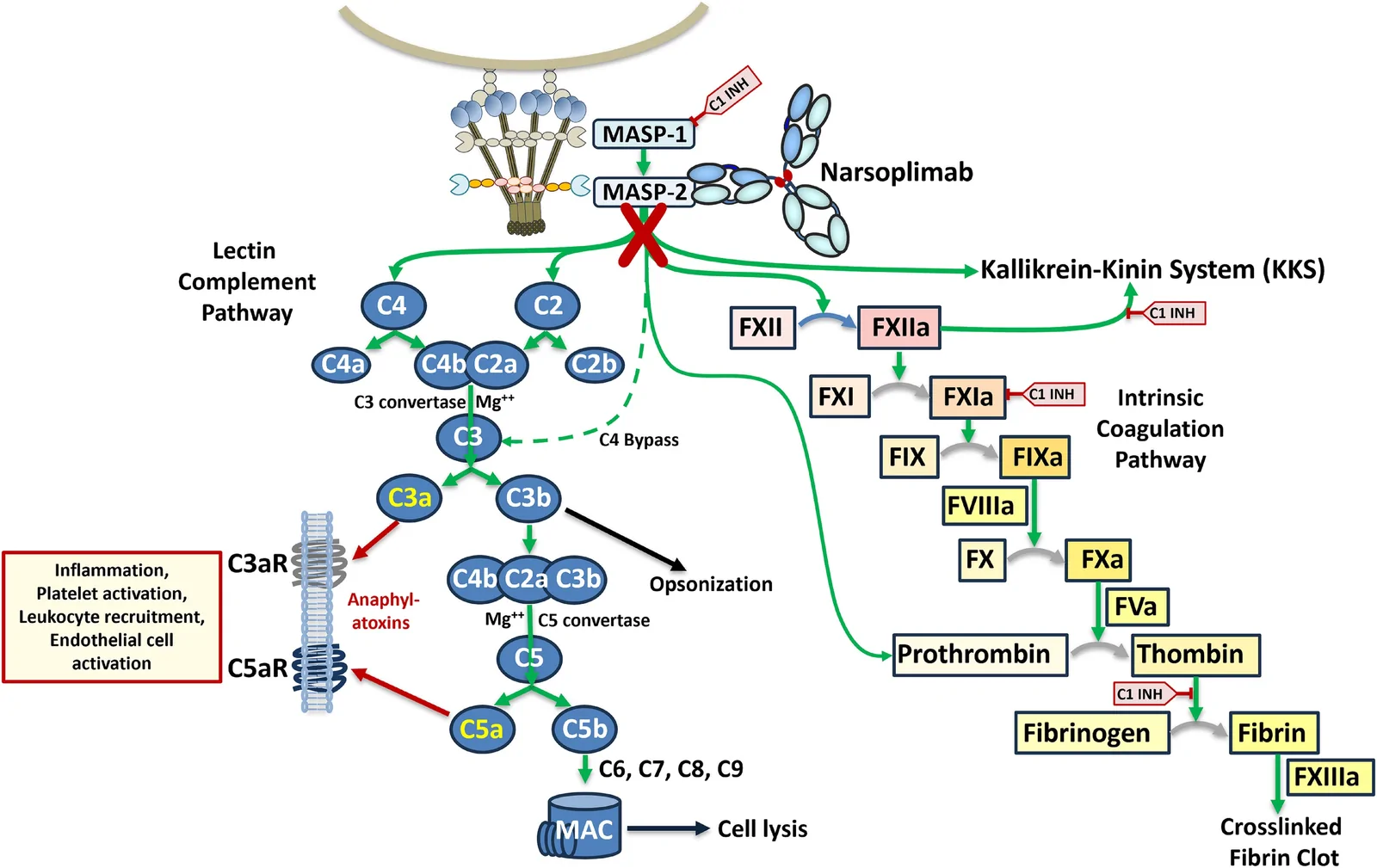
Activation of two pathways, the lectin complement pathway and the intrinsic coagulation pathway by MASP-2. Upon damage to the vascular endothelium, the zymogen Factor XII (FXII) is activated to FXIIa, which then activates two pathways, the lectin complement pathway (left) and the intrinsic coagulation pathway (right). Narsoplimab binds and neutralizes the enzymatic activity of MASP-2 to block the downstream effects of both pathways. Left: Lectin complement pathway in which Mannose-Binding Lectin (MBL) binds a sugar array on the surface of a cell with MASP-1 and MASP-2 in association. Binding ligand causes MASP-1 to self-activate and then active MASP-2, which activates C4 and C2 to C4b and C2a (both are the larger cleavage products; note that C2a has been called “C2b” in the older literature [293]), respectively. C4b/C2a in the presence of Mg^++^ function together as C3 convertase, which cleaves C3 into the anaphylatoxin C3a and C3b, which can opsonize microorganisms. C3b also interacts with the C3 convertase (C4b/C2a) and Mg^++^ to cleave C5 into the anaphylatoxin C5a and C5b, which recruits C6–C9 to form the membrane attack complex (MAC) that forms pores in membranes causing lysis [293, 295, 296]. C1-INH only inhibits this pathway at MASP-1, as noted with the boxed arrow. Right side: FXIIa activates Factor XI (FXI) to FXIa, which then continues the activation factors in the coagulation cascade until fibrinogen is converted to the insoluble fibrin mesh to promote clot formation [276, 277]. As noted with boxed arrows, C1 inhibitor (C1-INH) also inhibits this process at multiple sites, including FXII activation, FXIIa activation of FXI, and the terminal reactions in the common coagulation pathway. The green arrows indicate feedback activation steps.

When a PRM binds to a pathogen, it triggers the activation of associated enzymes known as MBL-associated serine proteases (MASPs). MASP-1 and MASP-2 form complexes with PRMs and attachment of the PRM to the pathogen’s surface triggers the self-activation of MASP-1 [295]. Once MASP-1 is activated, it cleaves and activates MASP-2, which serves as the essential “effector” enzyme driving the rest of the pathway [295]. Activated MASP-2 then cleaves the complement proteins C4 and C2 into C4b and C2a [296], respectively, which combine on the pathogen’s surface to form C3 convertase (C4b2a). From the C3 convertase stage onward, the lectin pathway is identical to the classical pathway [293]. The C3 convertase cleaves C3 into C3a (an inflammatory signal) and C3b, an opsonin that “tags” the pathogen for destruction [293]. C3b binds to the C3 convertase to form the C5 convertase (C4b2a3b), which cleaves C5. The final steps lead to the formation of the membrane attack complex (MAC), which forms pores in the pathogen’s membrane, causing it to lyse [293] (Fig. 5).

MASP-2 also acts as a bridge to the coagulation system by cleaving prothrombin to produce thrombin. While its primary role is cleaving C4 and C2 to initiate complement activation, it can also directly activate Factor XII (FXII), triggering the contact pathway of coagulation and the pro-inflammatory KKS (Fig. 5) [297, 298]. Activated MASP-2 can cleave the single-chain zymogen FXII into its active two-chain form, FXIIa. This activation contributes to “thromboinflammation,” wherein the complement and coagulation cascades amplify each other [297]. FXIIa subsequently activates prekallikrein (leading to bradykinin release) and Factor XI (initiating the intrinsic clotting pathway). Inhibiting MASP-2 effectively reduces the downstream activation of Factor XII and its associated pathologies, including reduction in thrombosis and fibrin formation, and importantly, the generation of bradykinin, a potent mediator of swelling and pain in HAE [297, 298].

MASP-2 is a multi-domain enzyme that serves as the primary activator of the lectin pathway in the complement system [295]. It is composed of six distinct domains divided into two functional parts, one of which is the N-terminal segment (CUB1–EGF–CUB2) responsible for forming a homodimer and binding to PRMs [295]. The CUB1 and CUB2 domains enable calcium-dependent attachment to the collagen-like stalks of a PRM, while the epidermal growth factor (EGF)–like domain also includes a calcium-binding site [295]. The C-terminal segment (CCP1–CCP2–SP) acts as the enzyme’s catalytic section. The CCP1 and CCP2 domains are two complement control protein (CCP) modules that function as exosites, providing binding surfaces for large substrates such as C4. The serine protease (SP) domain is a chymotrypsin-like domain that cleaves C2 and C4 [295].

### Hematopoietic stem cell transplant–associated thrombotic microangiopathy

Allogeneic hematopoietic stem cell transplantation (allo-HSCT) is a medical procedure wherein a patient receives healthy hematopoietic stem cells from a donor to replace their own compromised or diseased bone marrow [299]. In contrast to autologous transplantation, which utilizes the patient’s own cells, allogeneic transplantation depends on a genetically distinct donor, often a matched sibling or an unrelated volunteer [299]. Allo-HSCT remains a pivotal curative intervention for a range of malignant and nonmalignant conditions, including: (i) hematological malignancies such as leukemias, lymphomas, and MM; (ii) disorders like aplastic anemia, characterized by insufficient blood cell production by the bone marrow; (iii) metabolic disorders with inborn errors of metabolism, such as Hurler’s syndrome and adrenoleukodystrophy; and (iv) genetic disorders including sickle cell disease, thalassemia, and primary immunodeficiency syndromes like severe combined immunodeficiency (SCID) [299]. Over 80 000 allo-HSCT procedures are performed worldwide annually across these various disorders [300].

Despite advancements in allo-HSCT procedures over the past decade, significant morbidity and mortality continue to limit its broader application [298]. One significant allo-HSCT-related adverse event is transplant-associated thrombotic microangiopathy (TA-TMA), which has increasingly been recognized as a major risk factor leading to increased morbidity and mortality [298]. TA-TMA is characterized by microangiopathic hemolytic anemia and thrombocytopenia, resulting from widespread endothelial dysfunction and complement activation, which lead to microthrombi formation [298]. The clinical manifestation of TA-TMA is highly variable and can affect any organ, although it most commonly impacts the kidneys and brain.

In a recent multicenter prospective study, it was demonstrated that ~57% of patients undergoing allo-HSCT developed TA-TMA within 100 days post-transplantation. Furthermore, nearly 22% of these patients were classified as having severe TA-TMA, which was correlated with a significantly elevated mortality rate [301].

The primary drivers for TA-TMA are described as the “three-hit hypothesis” [302], including: (i) Hit 1: underlying predisposition of a patient such as having aplastic anemia or having higher susceptibility to endothelial damage and/or complement activation; (ii) Hit 2: injury to the vascular endothelium by pretransplant conditioning (e.g. chemotherapy, radiation); and (iii) Hit 3: additional exogenous triggers such as infection due to immune suppression, formation of GVHD, or use of immunosuppressive medicines [302]. MASP-2 is a key mediator of TA-TMA via endothelial cell injury resulting in a dysregulated lectin complement pathway and its ability to initiate the coagulation cascade through its activation of FXII to FXIIa [302].

Prior to the approval of narsoplimab, there were no FDA-approved treatments specifically for treatment of TA-TMA [303]. Clinicians often employed various off-label medications and procedures to manage the condition. The most common off-label treatments used before 2025 were complement C5 inhibitors such as eculizumab and ravulizumab [303].

### Yartemlea® (narsoplimab-wuug)

Yartemlea® (narsoplimab-wuug), sponsored by Omeros Corporation, was approved by the FDA on 24 December 2025, for the treatment of TA-TMA [304]. Narsoplimab is the first drug ever to be FDA-approved for treatment of TA-TMA, although as mentioned above, anti-C5 antibodies have been used off-label for some patients [303]. Narsoplimab is a first-in-class anti-MASP-2 fully human IgG4λ with a stabilized hinge (S228P) [67, 68] that binds and neutralizes MASP-2, a pathway activating protein that is elevated in thrombotic angiopathies, of which TA-TMA is one [305]. By inhibiting MASP-2, narsoplimab blocks the initiation of both the lectin pathway of complement activation, preventing endothelial damage [306, 307], and MASP-2-mediated activation of the intrinsic coagulation cascade, reducing the risk of small blood vessel micro-thrombosis, a characteristic of TA-TMA [307]. It is important to note that MASP-2 inhibition by narsoplimab leaves intact the lytic arm of the classical pathway of complement, which reduces the risk of infection, particularly with encapsulated microorganisms such as *Neisseria meningitidis* [308].

The efficacy of narsoplimab was evaluated in a single-arm, open-label clinical study (TA-TMA Study) involving 28 patients [303] (NCT02222545 [309]), supplemented by data from 19 additional patients (6 pediatric and 13 adult) enrolled in an expanded access program (EAP; NCT04247906 [311]). In the TA-TMA Study, patients received weekly IV doses of narsoplimab of either 4 mg/kg for patients ≤50 kg or 370 mg for patients >50 kg. The primary efficacy endpoint in the TA-TMA Study was the TMA response, defined as an improvement in both laboratory TMA markers (e.g. lactate dehydrogenase [LDH] and platelet counts) and either an enhancement in organ function or independence from the need for further transfusions. A complete TMA response was observed in 61% (17/28) of the narsoplimab-treated patients. In the expanded access program (EAP) (NCT04247906 [311]), a complete TMA response was achieved in 68% (13/19) of evaluable patients. Patients treated in both the primary TA-TMA study and the EAP exhibited a 100-day survival rate from the time of diagnosis of ~73%–74%, which is significantly superior to the 10% survival rate observed in historical controls [310], and a median OS of 274 days [303]. Treatment with narsoplimab across all study cohorts resulted in a three- to four-fold reduction in mortality risk [303]. Additionally, disease markers, including platelet count, haptoglobin, and LDH, showed improvement in patients treated with narsoplimab [310].

Narsoplimab has a slightly higher volume of distribution at 10.9 L [64] than most IgGs (typical range is 3–8 L [312]), indicating that it was distributed not only throughout circulation but also into some extravascular space. The circulating half-life for narsoplimab was a modest 8.7 days, and the clearance was relatively high at 2.88 L/d [64]. These data indicate why the dosing schedule is QW [64]. In a small clinical cohort, only 3/28 (11%) of patients had ADAs against narsoplimab, and only one of those was found to be neutralizing. There did not appear to be any effect on either PK or efficacy of narsoplimab due to the ADAs [64].

## Imaavy® (nipocalimab-aahu) – anti-FcRn for myasthenia gravis

### FcRn

FcRn plays a crucial role in maintaining the balance of endogenous IgG, significantly contributing to the extended (on average, ca. 19 days [3]) half-life of circulating human IgG1, IgG2, and IgG4 isotypes, as well as human serum albumin (HSA) [313]. This is achieved by FcRn binding to pinocytosed IgG at an acidic pH, shielding it from lysosomal degradation, and then transporting the bound IgG back to the cell surface, where it is released back into circulation at a neutral pH [313]. FcRn shares a high degree of similarity with MHC class I receptors; however, FcRn lacks the peptide binding groove found in other MHC class I receptors, and it does not participate in Class I presentation. FcRn is widely expressed on cells of the reticuloendothelial system, such as endothelial and epithelial cells, hepatocytes, and myeloid cells, as well as in the cells of most organs (e.g. lung, stomach, liver, kidney, bladder, muscle, skin) and other tissues [313]. Human FcRn beta-2-microglobulin (β2m) and α1 domains interact with the CH2 and CH3 domains of human IgG1,2,4 isotypes in such a way that the Fab arms of the IgG are positioned “upside down” close to the membrane [313]. This interaction takes place only in endosomes at a lower pH, such as pH 5–6, where the histidines H310, H433, and H435 in the Fc are charged, facilitating binding to residues E115 and D130 of FcRn. At neutral pH and higher (e.g. pH 7.4), the charge is lost, and FcRn’s ability to bind IgGs is nullified [313]. Because of its role in IgG homeostasis, there has been significant interest in FcRn as a potential target to reduce pathogenic IgG autoantibodies produced in autoimmune diseases [314], as detailed previously [11].

### Generalized myasthenia gravis

Dozens of autoimmune diseases are driven by the presence of autoantibodies targeting specific human proteins [315]. One of these, generalized myasthenia gravis (gMG), is characterized by the presence of pathogenic antibodies against either the acetylcholine receptor (AChR) or muscle-specific tyrosine kinase (MuSK) [316]. Historically, diseases featuring pathogenic autoantibodies were managed using high-dose IVIG [317], but until the past 5 years or so, there were limited drug-specific treatment options.

Over the past several years, it has been demonstrated that antagonism of the antibody recycling receptor, FcRn, lowers the overall IgG concentration in blood, and with that, lowers the concentration of pathogenic antibodies [314]. The initial agent approved for FcRn modulation was Vyvgart® (efgartigimod alfa), which received approval from the US FDA in 2021 for treating gMG in patients with anti-AChR antibodies [318]. Efgartigimod alfa is a 51 kDa IgG1-based mutated (M32Y, S34T, T36E, H213K, N214F) Fc fragment that competes with human FcRn and reduces overall serum IgG levels [319]. Rystiggo® (rozanolixizumab-noli), an anti-FcRn IgG-based antibody sponsored by UCB, was approved by the FDA in 2023 for treatment of gMG, making it the second FcRn targeted treatment option. Rozanolixizumab demonstrated significant efficacy in treating gMG by reducing pathogenic antibodies by 78% [320], comparable to plasma exchange procedures, IVIG treatment, and efgartigimod alfa [318]. Among the various FcRn-inhibiting therapies, it seems they generally lower circulating IgG levels by 25%–50% at effective doses [321]. This reduction appears to be sufficient to suppress pathogenic IgG immune complex–mediated immune responses [322].

### Imaavy® (nipocalimab-aahu)

Imaavy® (nipocalimab-aahu), which binds FcRn and blocks IgG recycling, was approved on 29 April 2025 by the FDA for treatment of adults and pediatric patients (12 years of age and older) with gMG who are positive for anti-AChR or anti-muscle-specific kinase (MuSK) antibodies [32, 33]. Additionally, in March 2025, the FDA granted nipocalimab Fast Track Designation (FTD) for the treatment of adult patients with moderate-to-severe Sjögren’s disease (SjD), following on the Breakthrough Therapy Designation (BTD) granted in 2024 for the investigational therapeutic. There are currently no advanced biologic therapies approved to treat SjD, although many potential therapeutics are in development.

Nipocalimab is a human IgG1λ mAb possessing a mutation N297A (Eu numbering [66]) of the *N*-glycosylation site in CH2 to make the antibody non-*N*-glycosylated [31]. Nipocalimab was originally discovered at Momenta Pharmaceuticals, which was then acquired in October 2020 by Johnson and Johnson, who developed and sponsored the drug for approval [32, 33].

Nipocalimab binds to both the FcRn β2m and α1 subunits at an epitope that provides direct competition with IgG Fc binding in recycling mode. The nipocalimab-mediated buried surface area on the FcRn-α chain, contributed by both VH and VL residues, is 879.6 Å^2^, whereas the area buried on the N-terminal residues of the β2M subunit is 137.9 Å2, primarily due to VL CDR1 [323]. Nipocalimab binds FcRn with high affinity at both neutral (*K*_D_, ca. 32 pM) and acidic (*K*_D_, ca. 58 pM) pH, which results in the significant reduction of circulating IgG, including pathogenic IgG antibodies [323]. The nipocalimab epitope does not, however, overlap with HSA epitope, and experiments confirmed that nipocalimab does not disrupt albumin binding or recycling [323]. Likely due to the fact that it is not *N*-glycosylated at N297 (Eu numbering [66]), nipocalimab does not possess significant ADCC, antibody-dependent cellular phagocytosis, or complement-dependent cytotoxicity, whereas an A297N glycosylated mutant of nipocalimab possessed all three functions [323].

Nipocalimab was approved largely on the results of “Study 1” (aka Vivacity-MG3) [324] (NCT04951622 [325]), a 24-week long Phase 3, multicenter, 1:1 randomized, double-blind, placebo-controlled clinical trial to evaluate the efficacy, safety, PK, and PD of nipocalimab administered to adults with anti-AChR or anti-MuSK antibody-positive gMG [32, 324, 325]. For this study, 98 patients each received nipocalimab or placebo, respectively. Nipocalimab was given as a loading dose of 30 mg/kg IV, followed by 15 mg/kg IV maintenance doses Q2W. The primary endpoint for nipocalimab efficacy was based on a comparison of the mean change from baseline to Week 24 in MG-ADL total score (functions of eight signs and symptoms typical of gMG) for nipocalimab-treated versus placebo cohorts [32, 324]. At week 24, patients treated with nipocalimab had a statistically significant improvement in MG-ADL total score over those given placebo (−4.7 vs −3.3; *P* = .002). A secondary endpoint was the efficacy of nipocalimab using the QMG total score, a 13-item grading system assessing muscle weakness, in which patients treated with nipocalimab had a statistically significant QMG total score as compared with those receiving placebo (−4.9 vs −2.1; *P* < .001) [32, 324].

Nipocalimab exhibited a low volume of distribution at 2.67 L, below the average of most antibodies [312], a mean terminal half-life of 29 h, and a clearance of 1.5 L/d [32], requiring very high dosing (30 mg/kg loading, 15 mg/kg maintenance) to achieve Q2W. ADAs were detected in 48% (49/102) of patients receiving nipocalimab, 19 (38.8%) of which were neutralizing. No effect was observed of ADAs on PK, PD, efficacy, or safety [32].

## Anniko® (penpulimab-kcqx) anti-PD-1 for NPC

### PD-1 checkpoint target

As described last year, the checkpoint receptor programmed cell death protein-1 (PD-1, also referred to as PCD1, CD279) is part of the CD28 receptor family and is expressed on activated T- and B-lymphocytes [9]. The ligands for PD-1, PD-L1 (also known as B7-H1, CD274) and PD-L2 (B7-DC) [125], are expressed on antigen-presenting cells (APCs) [326]. Ligation of either PD-L1 or PD-L2 with PD-1 functions to tamp down T-cell-mediated immune responses, including T-cell proliferation and cytokine production, to avoid excessive immune activation that may result in autoimmune responses [327]. The significance of this pathway lies in the fact that many types of cancer cells have hijacked the pathway by overexpressing PD-L1 to suppress anti-tumor T-cell responses [328]. PD-1 binds to the N-terminal domain of PD-L1 with a natural 1:1 affinity of 8.2 µM, and the interaction covers a buried surface area of 1970 Å^2^ [329]. Most anti-PD-1 and anti-PD-L1 antibodies developed and approved for cancer treatment are sub- to low-nanomolar binders to PD-1 or PD-L1 [329], making them strong competitors against the natural ligands. Furthermore, all FDA-approved anti-PD-1 and anti-PD-L1 antibodies have epitopes that overlap with the binding sites of the ligand, PD-L1, and the receptor, PD-1, respectively [9].

The first FDA-approved anti-PD-1 antibody was Merck’s Keytruda® (pembrolizumab), approved in September 2014 for metastatic melanoma treatment [330]. Pembrolizumab is now approved in the USA for 20 different indications, with global sales in 2025 alone nearing $32 billion [4]. Opdivo® (nivolumab), an anti-PD-1 mAb developed by Bristol-Myers Squibb (BMS), received FDA approval 3 months later (December 2014) for metastatic melanoma treatment [331]. Including penpulimab described in this section, there are now eight anti-PD-1 antibodies and four anti-PD-L1 antibodies that have received approval from the US FDA for treatment of a broad range of cancers [9]. Additionally, at least another 10 PD-(L)1 antibodies have been approved in China that have not yet received US FDA approval [332]. By the end of 2025, the combined market for PD-1/PD-L1 therapies has been valued at ~$60.4 billion annually in global sales [4], accounting for ~19% of all antibody sales worldwide (Fig. 2), approximately the same percentage as in 2024 [22] but slightly up from the 16% total market share in 2023 [11, 21]. This market is anticipated to maintain its leading position, with at least 30 mono- or bispecific anti-PD-(L)1 antibodies currently in advanced (Phase 3) clinical trials [1], many of which are expected to seek approval in the coming years.

### Nasopharyngeal carcinoma

Nasopharyngeal carcinoma (NPC) is a relatively rare form of cancer that affects the upper throat (nasopharynx) [333]. NPC is highly associated with infection by Epstein–Barr virus (EBV), with nearly 100% of nonkeratinizing cases of NPC being positive for EBV [333]. Although at least 90% of the worldwide population is infected with EBV, only certain populations, particularly those in Southeast Asia, are at risk for EBV infections developing into NPC [333].

Locally advanced metastatic or recurrent NPC is relatively rare, accounting for 0.7% of all cancers with 133 354 new cases globally in 2020, it is known for its malignancy potential [334]. The incidence of NPC is notably higher in Asian populations, especially among Chinese individuals, who represent over 70% of new cases [334]. Loqtorzi®, in combination with cisplatin and gemcitabine, was previously approved for use as first-line therapy, and as a monotherapy in later treatment stages [335, 336].

### Penpulimab approval for treatment of NPC

Anniko® (安尼可 in China; penpulimab-kcqx), developed by Akeso Biopharma Co., Ltd., was granted full approval by the FDA on 23 April 2025 for use with cisplatin or carboplatin and gemcitabine as a first-line treatment for adults with recurrent or metastatic nonkeratinizing, locally advanced NPC [30]. Additionally, penpulimab was approved for second-line or later-line treatment as a single agent for disease progression on or after platinum-based chemotherapy. Penpulimab had previously received approval for multiple oncology indications in China, starting in 2021 [337], and had $46 million in sales in 2025 [4].

Penpulimab (originally known as AK-105) is a humanized IgG1κ, with the mutations L234A, L235A, and G236A (Eu numbering; [66]) to significantly reduce interactions with Fcγ receptors [338]. As noted previously [9], even though both pembrolizumab and nivolumab possess human IgG4 isotype Fcs that have the ability to bind FcγRI, it is currently believed that binding to FcγRs is not required for optimal anti-PD-1 activity, and may be a liability with certain anti-PD-1 antibodies [339–341].

Penpulimab exhibits a high 1:1 affinity for PD-1, with a dissociation constant (*K*_D_) of 0.59 nM. More importantly, it demonstrates a significantly slower dissociation rate from the PD-1 receptor (9.51 × 10^−5^/s) as compared with pembrolizumab (2.8 × 10^−4^/s) and nivolumab (2.43 × 10^−4^/s) [338]. This reduced dissociation rate is attributed to its distinctive binding interactions with the glycosylated N58 residue on the BC loop of the PD-1 receptor [338]. Consequently, this slower off-rate results in a relatively higher receptor occupancy, ranging from 80% to 100% at steady state, thereby enhancing T-cell activity [338].

FDA approval of penpulimab for the treatment of NPC was primarily based on two clinical studies, Study AK105-304 [342] (first-line combination; NCT04974398 [343]) and Study AK105-202 [344] (monotherapy; NCT03866967 [345]). Study AK105-304 was a randomized, double-blind, multicenter trial in which 291 patients with recurrent/metastatic NPC who had not received prior chemotherapy were evaluated. Patients received 200 mg penpulimab IV or placebo, in combination with either cisplatin or carboplatin and gemcitabine, Q3W [343]. The primary end point in the AK105-304 clinical trial was PFS, with OS as the secondary end point, both as compared with placebo with cisplatin or carboplatin and gemcitabine. Median PFS in the penpulimab arm was 9.6 months (95% CI, 7.1–12.5 months), compared with 7.0 months (95% CI, 6.9–7.3 months) in the placebo arm, as well as an ORR of 68.1% [342]. After 12 months, 31% of patients in the treatment arm vs 11% of patients in the placebo arm were alive and progression-free [342]. The recommended dosing for combination therapy was 200 mg of penpulimab Q3W [29].

The efficacy of penpulimab as a monotherapy was determined in the open-label, multicenter, single-arm clinical trial, Study AK105-202 [344, 345], which studied 125 patients with unresectable or metastatic nonkeratinizing NPC whose disease had progressed postplatinum-based chemotherapy and one or more other lines of therapy. Patients were dosed IV with 200 mg of penpulimab Q2W or Q3W for a maximum of 24 months or until their disease progressed or they experienced unacceptable toxicity [344]. The primary end points were ORR and DOR, as evaluated by RECIST v1.1. Results were 28% (95% CI, 20%–37%) ORR and 46% DOR at 12 months (mDOR was not reached by the end of the analysis [344]). Based on the results, dosing penpulimab at 200 mg Q2W was recommended for monotherapy.

Penpulimab had a terminal half-life of 32 d and a clearance of 0.2 L/d, supporting both the Q2W- and Q3W-recommended dosing regimens [29]. About 24%–30% of patients receiving penpulimab either as monotherapy of in combination with cytotoxic drugs showed ADA responses. Of those patients with ADA responses, 17% (18/105) in the monotherapy group and 44% (39/88) in the combination therapy study had neutralizing antibodies [29]. It was not determined in those studies whether any of the ADA responses altered activity or pharmacokinetics of penpulimab [29].

## New hyaluronidase coformulations

The 2025 approvals of Keytruda QLEX® and Rebryvant Faspro® (Table 1) added to the eight previously approved antibody–hyaluronidase combinations [9] make it a total of 10 antibody-like biologics to be combined with a hyaluronidase for SC dosing.

The US FDA approved Keytruda QLEX® (pembrolizumab berahyaluronidase alfa-pmph; also known as MK-3475A) on 19 September 2025 for subcutaneous delivery of the anti-PD-1 antibody, Keytruda®, for treatment of 18 cancer indications [48–50]. Keytruda®, which is delivered via the IV route of administration, is losing its patent protection in 2028, after approval in 2014 as the first anti-PD-1 antibody for cancer treatment [346]. Considering that in 2024, Keytruda® accounted for $29.5 billion in sales comprising 45% of Merck’s total sales of ~$64.2 billion [347], this is a significant challenge for the company. The approval of Keytruda QLEX® all but guarantees that the Merck Keytruda anti-PD-1 antibody franchise will maintain a competitive sales and market position over the next decade. This is particularly important for Merck, given that the two main competitors for Merck’s Keytruda®, Opdivo® (nivolumab, anti-PD1) and Tecentriq® (atezolizumab, anti-PD-L1), already had achieved FDA approval in 2024 for subcutaneous formulations using Halozyme’s rHuPH20 (hyaluronidase; Enhanze®) [9, 50].

The hyaluronidase incorporated into Keytruda QLEX® was not rHuPH20, the recombinant human hyaluronidase developed under the Enhanze® technology portfolio as Hylenex® PH20 by Halozyme [348]. Instead, the hyaluronidase component of Keytruda QLEX® has been modified to increase the efficiency of delivery [349, 350] and was therefore recognized by the FDA as a novel biologic [6]. The mutated version of hyaluronidase, called berahyaluronidase alfa, was developed by the Korean company, Alteogen [350, 351] and licensed to Merck as ALT-B4 [352]. The sequence of berahyaluronidase alfa ALT-B4, also known as ALT-BB4 or Tergase® (brand name) as a stand-alone product [350], is shorter than PH20 by 16 residues (two off from N-terminus and 14 residues shorter at C-terminus), and has modifications in 15 residues in the region of residues 304–324 (304-SWENTRTKESCQAIKEYMDTT-324) [353].

The mean bioavailability for pembrolizumab after delivery as pembrolizumab berahyaluronidase alfa-pmph in a recent set of clinical trials was 61% [349], ~10% lower as compared with 74% and 72% for Opdivo Qvantig® and Tecentriq Hybreza®, respectively [9]. The incidence of ADAs emerging from treatment with pembrolizumab berahyaluronidase alfa were 1% and 2% for pembrolizumab and berahyaluronidase, respectively [349, 350], and injection site reactions occurred in 16% of patients treated, all of which were limited to grades 1 or 2 [349, 350].

The recommended dosing for Keytruda QLEX® is an initial Q3W dosing regimen (395 mg pembrolizumab/4800 units hyaluronidase in 2.4 ml) SC in ~1 min, followed by a Q6W dosing schedule (790 mg/9600 units; 4.8 ml) SC for ~2 min [48]. This dosing regimen is quite favorable compared with Opdivo Qvantig®, which is dosed for several indications Q2W (600 mg nivolumab and 10 000 units hyaluronidase in 5 ml) SC for ~3–5 min or Q4W (1200 mg atezolizumab/20,000 units hyaluronidase in 10 ml; two vials worth) SC for ~6–10 min [354]. Similarly, the anti-PD-L1 antibody, Tecentriq Hybreza®, is dosed every Q3W (1875 mg atezolizumab and 30 000 units hyaluronidase in 15 ml) SC for ~7 min [355].

Separately, the US FDA approved Rybrevant Faspro® on 17 December 2025 for subcutaneous delivery of the previously approved anti-EGFR × cMET bispecific antibody, amivantamab, for treatment of EGFR-mutated NSCLC [61, 62]. Rybrevant Faspro® utilizes the same hyaluronidase (HP20), the ENHANZE® delivery technology licensed from Halozyme, that has been used in previous SC formulations [9]. Rybrevant Faspro® requires only ~5 min to be administered, as compared with the original Rybrevant IV infusion, which required 2–8 h of infusion [61]. Additionally, a five-fold reduction in administration-related reactions was observed in clinical trials (13% for RF vs 66% with IV Rybrevant) [61]. Additionally, the incidence of venous thromboembolism was lower with the subcutaneous form (11%) compared to the IV arm (18%) [61].

## Lerochol® (lerodalcibep-liga)–anti-PCSK9

### PCSK9

PCSK9 is a protein primarily produced in the liver that plays a critical role in regulating blood cholesterol levels. It works by controlling the number of low-density lipoprotein receptors (LDLRs) on the surface of liver cells, which are responsible for clearing “bad” cholesterol from the bloodstream [356]. FDA-approved mAbs, such as Praluent® (alirocumab) and Repatha® (evolocumab), prevent PCSK9 from binding to the LDL-R, enabling a more rapid recycling of the LDL-R, which significantly reduces LDL-C due to enhanced clearance, even in subjects with heterozygous familial hypercholesterolemia (HeFH) [357].

### Heterozygous familial hypercholesterolemia

HeFH, an autosomal semi-dominant genetic disorder that affects 25–30 million people worldwide, is characterized by elevated low-density lipoprotein cholesterol (LDL-C) levels from birth [358]. HeFH results from dysfunctional variants in genes responsible for the clearance of LDL-C, typically due to loss-of-function variants in the low-density lipoprotein receptor gene (LDLR) or, less commonly, the apolipoprotein B gene (APOB) [359]. Alternatively, gain-of-function variants in the PCSK9 gene can also cause HeFH [359, 360].

### Lerodalcibep-liga (Lerochol®)

Lerochol® (lerodalcibep-liga), which was developed by LIB Therapeutics (Cincinnati, OH), was approved by the FDA on 12 December 2025 for the treatment of adults with high low-density lipoprotein cholesterol (LDL-C), including individuals with HeFH [55, 56]. Lerodalcibep (formerly LIB-003) is composed of a high-affinity anti-PCSK9 nonantibody-binding domain, known as an adnectin [361], derived from human tenth fibronectin type III (FN3) domain, fused via a “GSGSGS” peptidyl linker to the C34A mutant of HSA [54]. Lerodalcibep is not an antibody but is worthy of “honorable mention” due to its antibody-like mechanism of action and differentiation from antibody competitors in the field. The fusion protein is 77 kDa in size, with the N-terminal adnectin portion composed of 96-amino acid residues [54].

The FDA’s approval of lerodalcibep was predicated on findings from five global phase 3 registration studies, collectively referred to as the LIBerate program, which encompassed 2900 patients. A pertinent example is the LIBerate-HoFH phase 3 trial [362] (NCT04798430 [363]), which conducted a head-to-head comparison of lerodalcibep and evolocumab for the treatment of homozygous familial hypercholesterolemia (HoFH). Patients with genetically confirmed HoFH were randomly allocated to receive either lerodalcibep 300 mg QM or evolocumab 420 mg QM for a duration of 6 months, followed by a 2-month washout period before switching to the alternate treatment for an additional 6 months [362, 363]. The primary efficacy endpoint was the percentage change from baseline in LDL cholesterol concentration at Week 24 for both dosing periods. LDL cholesterol levels were reduced from a baseline of 10.59 mmol/L (SD, 4.37) by −4.9% with lerodalcibep compared to −10.3% with evolocumab. When averaged across all monthly visits, the LDL cholesterol response was −9.1% with lerodalcibep and −10.8% with evolocumab [362].

Lerodalcibep is self-administered as a high-concentration/small-volume (300 mg in 1.2 ml) SC injection. Clearance of free lerodalcibep-liga is 0.36 L/d, with an estimated half-life of ~10 days, which supports the QM dosing [55]. The most striking feature of lerodalcibep is that it has extended room-temperature stability, for up to 3 months, giving patients expanded freedom to administer the drug SC at their time and place of choice [55, 56]. This small, novel antibody-mimetic biologic is a direct competitor to alirocumab and evolocumab both of which are commercial mAbs that bind and neutralize PCSK9, but both of which require refrigeration.

## Summary

In 2025, 10 new antibody-based biologics were approved by the US FDA. One key factor that stands out is the approval of two new ADCs, bringing the total of FDA-approved ADCs to 14. Additionally, two antibodies with the half-life extending YTE mutations were approved in 2025, bringing the total of FDA-approved half-life extended antibodies to eight (includes the covid antibodies that were granted EUAs). Considering the large number of antibodies in late-stage and/or registrational clinical trials [4], we should expect to see another large group of novel antibodies approved by the US FDA in 2026.

## Data Availability

Data supporting the information provided here are derived from the public literature or public websites.
